# Improving magnetic field homogeneity in prostate MR imaging and spectroscopy using an add-on local external shim coil array

**DOI:** 10.1007/s10334-025-01290-y

**Published:** 2025-09-11

**Authors:** Angeliki Stamatelatou, Carlijn Tenbergen, Sahar Nassirpour, Paul Chang, Jack van Asten, Arend Heerschap, Tom Scheenen

**Affiliations:** 1 Department of Medical Imaging, (766), Radboud University Medical Center, Geert Grooteplein 10Radboudumc, P.O. Box 9101, 6500 HB Nijmegen, the Netherlands; 2 MR Shim GmbH, Ferdinand-Lassalle-Straße 36, 72770 Reutlingen, Germany

**Keywords:** Prostate, B_0_ shimming, Proton MR spectroscopy and spectroscopic imaging, Diffusion-weighted imaging

## Abstract

**Objective:**

To improve B_0_ field homogeneity in prostate MR imaging and spectroscopy using a custom-designed 16-channel external local shim coil array**.**

**Methods:**

In vivo prostate imaging was performed in seven healthy volunteers (mean age: 40.7 years) without bowel preparation. A simulation study determined the most robust configuration of the add-on shim coil together with shim coils from the MR system. The combination of the add-on shim coil array with 1st-order shims of the MR scanner was used and compared to the MR system’s reference standard of conventional 1st and 2nd-order shimming. Performance was evaluated using the standard deviation (SD) of off-resonance frequencies (ΔB_0_) from B_0_-maps, the distortion of diffusion-weighted images (DWI) by the Dice similarity coefficient (DSC) with reference to T_2_W images, and the citrate linewidths of ^1^H-MR spectroscopic imaging (^1^H-MRSI) of the prostate.

**Results:**

Overall, improvements were made with the combination of coils, with DSC rising from 0.90 ± 0.02 to 0.92 ± 0.02 (*p* = 0.01), a reduction in the SD of ΔB_0_ from 18.5 ± 4.7 Hz to 16.5 ± 3.6 Hz, and a decrease in citrate linewidths from 8.3 ± 5.7 Hz to 7.9 ± 3.6 Hz (*p* = 0.018), all compared to the reference standard of conventional 1st and 2nd-order shimming.

**Conclusion:**

A dedicated external shim coil array can improve B0 homogeneity in the prostate, enhancing image quality and precision in multi-parametric MRI.

**Supplementary Information:**

The online version contains supplementary material available at 10.1007/s10334-025-01290-y.

## Introduction

The reliability and quality of MR examinations heavily rely on the uniformity of the main magnetic field (B_0_), as it directly impacts image quality, spatial registration, signal strength, and spectral resolution [1–4]. Clinical MRI systems are equipped with shim coils producing additional static magnetic fields with up to 2nd order spherical harmonic-shaped components to homogenize the main magnetic field in the organ or area of interest. For brain studies, additional shimcoils have been built in or around the radiofrequency receive headcoil [5–9]. In such setups, the additional shim coils are relatively close to the brain and they are in a fixed position with respect to the scanner’s existing shim coils in the isocenter of the MR system. In the body, and in particular in the prostate, an organ situated in the lower abdomen, achieving optimal magnetic field homogeneity is even more challenging. The presence of susceptibility differences between air and tissue, attributed to bowel gas and amplified by motion, imposes complex B_0_ field distortions, predominantly affecting the posterior region of the prostate [3, 10]. Adding additional local shim coils to the pelvis to influence magnetic field homogeneity in the prostate introduces two additional issues when compared to brain setups: add-on coils for the pelvis are further away from a small organ of interest (the prostate), and the relative position of anterior coils on top of the body varies, depending on the physique of the subject.

Echo-planar imaging (EPI) based diffusion-weighted imaging (DWI) and magnetic resonance spectroscopy (MRS) techniques employed in prostate imaging are particularly susceptible to B_0_ field inhomogeneities. In DWI, off-resonance effects can result in geometric distortions. Distortion correction methods based on spatially varying B_0_ field maps have been developed to address these challenges, providing effective correction schemes for warped EPI images, especially in the prostate [1, 11]. However, potential temporal changes in the B_0_ field resulting from physiological or patient motion [12] can introduce inaccuracies in pixel shifts across a DW dataset, hindering the accurate computation of apparent diffusion coefficient (ADC) maps for prostate cancer assessments [13]. In MRSI, off-resonance effects can cause spectral line broadening and frequency shifts, thereby compromising water suppression and introducing lipid contamination within prostate spectra [14, 15]. Temporal B_0_ field variations during acquisition can introduce small frequency shifts between signal averages in MRS, leading to incoherent averaging and spectral line broadening. This degrades spectral resolution, causes overlapping peaks, and reduces the accuracy of metabolite quantification [16]. Consequently, understanding and accounting for B_0_ field variations is crucial in prostate MRI [3, 4], for which additional degrees of freedom with a local add-on shim coil could be beneficial.

Existing approaches to improve B_0_ field uniformity in the prostate have mainly targeted susceptibility artifacts caused by rectal gas, particularly due to its paramagnetic oxygen content. Patient preparation techniques involve the administration of antispasmodic agents, such as hyoscine butylbromide (Buscopan, Boehringer, Ingelheim, Germany) or Glucagon (GlucaGen, Novo Nordisk A/S, Bagsvaerd, Denmark) to reduce bowel motion, and the insertion of rectal catheters [17] to evacuate gas. While these methods have shown some effectiveness in preventing artifacts [18], they do not specifically address the challenges associated with improving B_0_ field homogeneity in the prostate.

Various methods have been documented to correct induced artifacts from B_0_ field inhomogeneities during post-processing, mainly in the field of brain imaging. These post-processing techniques involve correcting spatial mis-registration [2, 19] but require additional reference data or extra scans [1, 20, 21], using the point-spread function [22, 23] or data acquired with phase-encoding gradients of opposing polarity to correct image distortion [24, 25]. It is important to note that despite these post-processing efforts, some signal loss remains irrecoverable [8].

During the acquisition phase, selecting suitable MR acquisition parameters can effectively reduce artifacts caused by magnetic field variations. A higher spatial resolution—SNR permitting—effectively mitigates field variations within a voxel, thereby decreasing signal dephasing. High-bandwidth RF pulses with large slice selection gradients will minimize displacement artifacts [26]. Increased acquisition bandwidths reduce spatial misalignment and image distortion [27, 28]. Additionally, both passive and active shimming techniques can be deployed. Passive shimming approaches involve correcting magnetic field inhomogeneities by strategically positioning magnetically susceptible materials within the scanner bore. This approach has limitations in adapting to experiment-specific conditions and varying shim requirements originating from differences in subject anatomy or placement. Active shimming, on the other hand, is the most common approach and involves B_0_ homogenization through correction fields generated by electrical coils. The conventional strategy for minimizing magnetic field variations using active shimming is to overlay magnetic fields characterized by spatial variations governed by spherical harmonic functions [29, 30]. Generally, the higher the maximum spherical harmonic order applied for B_0_ shimming, the more closely the shim field can approximate and compensate for the spatial characteristics of B_0_ field deviations. Numerous methodologies have been developed in this area over the years, primarily focusing on brain imaging applications [5–9, 31–33]. These methods include passive shimming using susceptibility materials, static active shimming with higher-order spherical harmonics, dynamic shimming strategies, and the use of local multicoil arrays for region-specific B0 optimization.

Motivated by the success of additional local shimming methods in the brain, in this pilot work we introduce the design, implementation, and analysis of the first dedicated external add-on local shim coil array specifically tailored to the prostate. The array consists of 16 circular, multi-turn shim coils, eight anterior and eight posterior, arranged to closely surround the prostate region while being integrated around the external receive coils. The purpose of this add-on shim coil is to improve B_0_ field homogeneity within the prostate, leading to enhanced image quality, reduced artifacts, resulting in improved accuracy in multi-parametric MRI. A simulation study to determine the most robust strategy for combining the add-on shim array with spherical harmonic shims is presented and finally the feasibility of this add-on shim array is assessed by analysis of B_0_-maps, EPI-based DWI, and ^1^H-MRSI.

## Methods

### 2.1 Hardware

All measurements were conducted using a clinical 3 T MR system (MAGNETOM Prisma-Fit, Siemens Healthineers, Erlangen, Germany) equipped with external multi-channel body and spine phased-array coils for signal reception.

The experimental setup featured a custom-designed 16-channel external add-on shim coil array, structured into four distinct "shim" modules (Dia, MR Shim GmbH, Reutlingen, Germany). The geometry, shape, and number of turns of the shim coils were selected to achieve maximum coverage while taking into consideration the available space and mechanical constraints of the MRI setup. The shim coils have a standard circular shape, and the number of turns and size (20 turns and 6 cm diameter) were chosen such that the shim coils can fit inside the shim holder while keeping the thickness of the shim holder below 1 cm, and to ensure that the required current for each channel remains below 1 A. The shim cables were made with 20 AWG wires as twisted pairs.

**Fig. 1:**
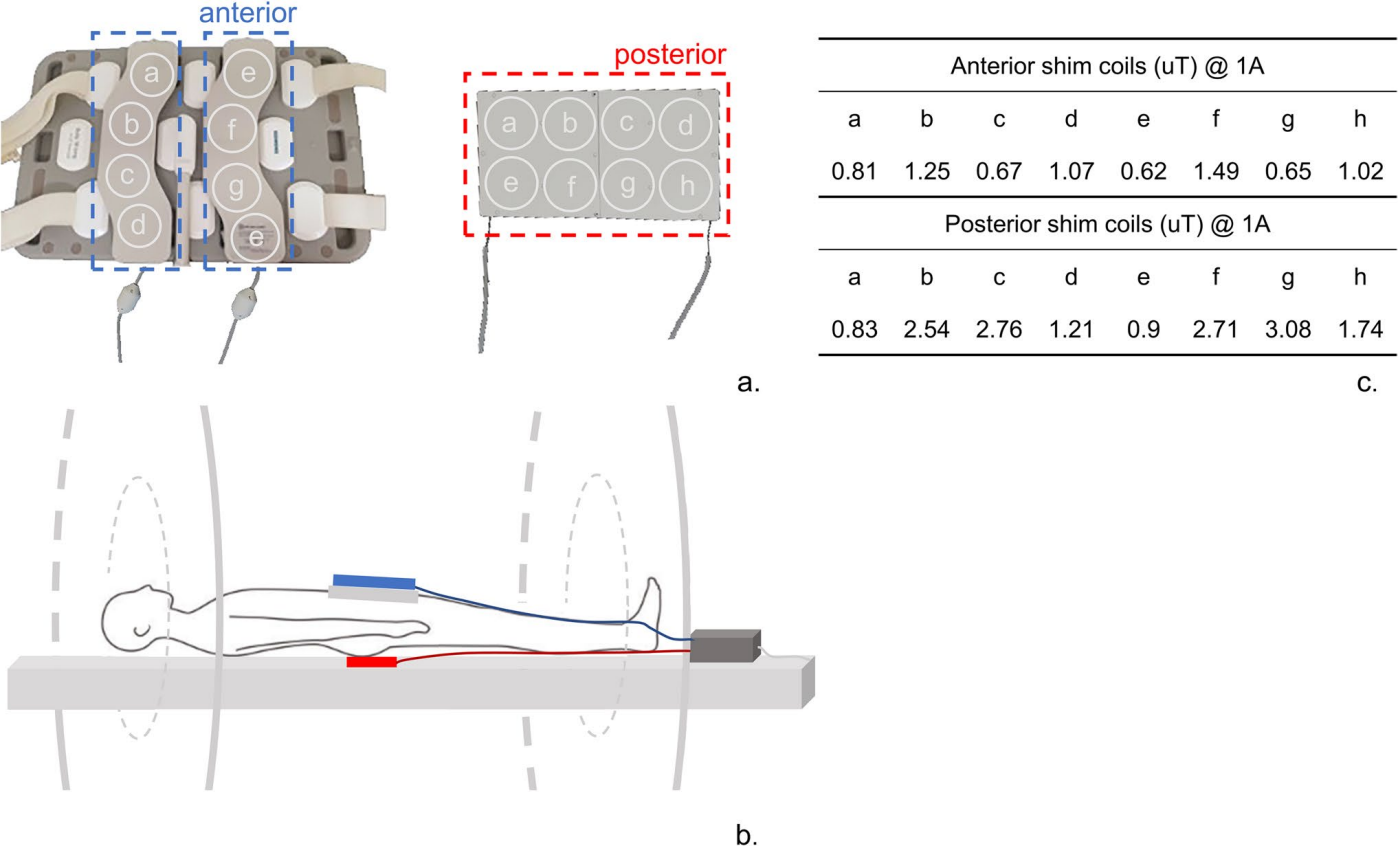
Experimental setup of an external shim coil array. **a** the 16-channel local shim coil array, consisting of two posterior modules (red frame) and two anterior modules (blue frame). The anterior modules are positioned on top of the external RF 18-channel body phased-array coil. **b** System configuration. The anterior shimming modules (blue) are positioned around the receive RF coil and the posterior modules (red) are placed under the mattress of the patient bed. The shim modules are connected to the filtered shim interface box. **c** the maximum field strengths of the coil elements observed in vivo within the prostate of a representative volunteer at a current of 1 A

The shim coil setup included eight channels on the anterior side, divided into two modules (blue frames in Fig. 1a), and eight channels on the posterior side, distributed across two modules (red frame in Fig. 1a), for full coverage. On the posterior side, the eight channels were uniformly distributed within the available space of the patient bed using a shim coil holder with a width of approximately 35 cm. The main patient mattress normally sits on top of the spine coil housing, so the positioning was achieved by placing the channels below the mattress but above the spine coil. To maximize coverage, the eight shim coils were arranged in two rows, resulting in a shim coil holder length of ~ 16 cm. For the anterior coils, the shim holder design was constrained by the shape of the body array receive coil, to which the coils were mechanically fixed. A configuration of two columns was chosen (four shim coils in each column in a shim holder covering a span of approximately 38 cm in the head-foot direction). To configure this setup, the anterior modules were positioned on the 18-channel RF body receive array coil, while the posterior modules were positioned underneath the patient's bed mattress on top of the RF spine-array coil, as indicated in Fig. 1b. Each rigid shim module had fiducial markers embedded in the housing, enabling the detection of the position of the shim coils for each subject.

A filtered shim interface box was positioned at the head of the scanner table. This interface box served as the central hub for the connection of the four shim modules. To drive the shim coils with electric currents, two amplifier units (Jupiter, MR Shim GmbH Reutlingen, Germany) were deployed. These amplifier units were positioned in the corner of the scanner room situated beyond the 100 Gauss line. Adjacent to the amplifier units, a non-magnetic Ethernet-to-fiber optic converter box was powered by a 5 V power adapter, which was also connected to the mains supply. The box was linked to the master shim amplifier unit through a short Ethernet cable, and to its counterpart outside of the magnet room positioned next to the host computer using a duplex fiber optic cable. This cable was threaded through the waveguide opening of the MRI room penetration panel, ensuring an interference-free connection. In the MRI control room, the Fiber optic-to-Ethernet converter box was powered using a 5 V power adapter and connected to the shim controller PC through an Ethernet cable.

**Fig. 2:**
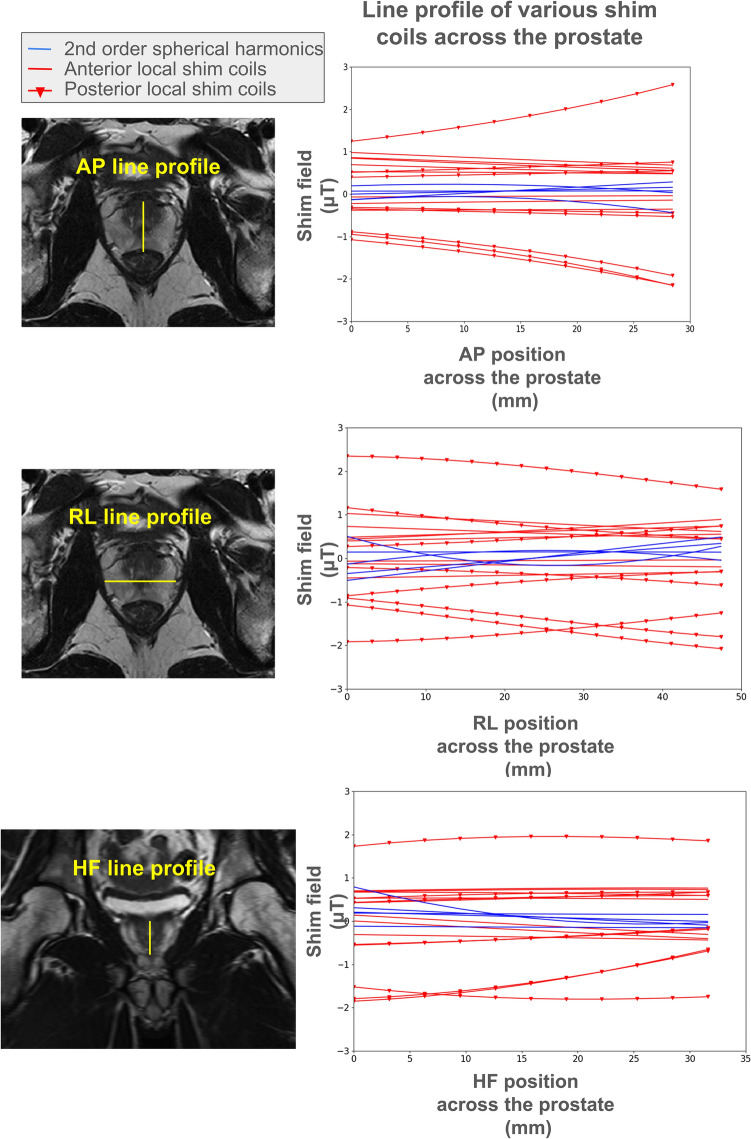
Strength and penetration depth of the local shim array. The line profile of the magnetic field created by each of the 16 local shim coils at 1A of shim current on a representative volunteer are plotted (red) for the head-foot (HF), anterior–posterior (AP) and right-left (RL) directions. As a comparison, the line profile of the magnetic fields created by scanner’s 2nd order spherical harmonic shim values set at 1000 μT/m2 is also shown (blue). The curves for the local shim coils illustrate changes in the order of microTesla over distances of 30 to 50 mm. Changes of 1 microTesla can result in a frequency difference of up to 40 Hz, and when multiple elements are combined, they can counteract inhomogeneities on the order of 150 Hz

Figure 2 illustrates the field patterns generated by each local shim coil across the prostate and B_0_ map distributions generated by each coil are shown in Supplementary Fig. 1. Depending on the position of each local shim coil relative to the prostate, the coils were able to produce a peak-to-peak variation of between 36 to 129 Hz over the extent of the prostate region. The maximum field strength produced by each of the local shim coils across the prostate for 1A of shim current is also summarized in the table of Fig. 1c.

### Acquisition protocol

#### Subjects

We examined seven healthy volunteers (V1 to V7), with a mean age of 40.7 years (range 28 to 65 years). These volunteers underwent scanning without any prior bowel preparation. Ethical approval for the study was obtained from the local ethical committees at Radboudumc, Nijmegen, the Netherlands.

#### Shimming procedures

All of the supplementary hardware described above was operated via a controller PC utilizing the *Arche* shimming software (MR Shim GmbH Reutlingen, Germany).

**Fig. 3:**
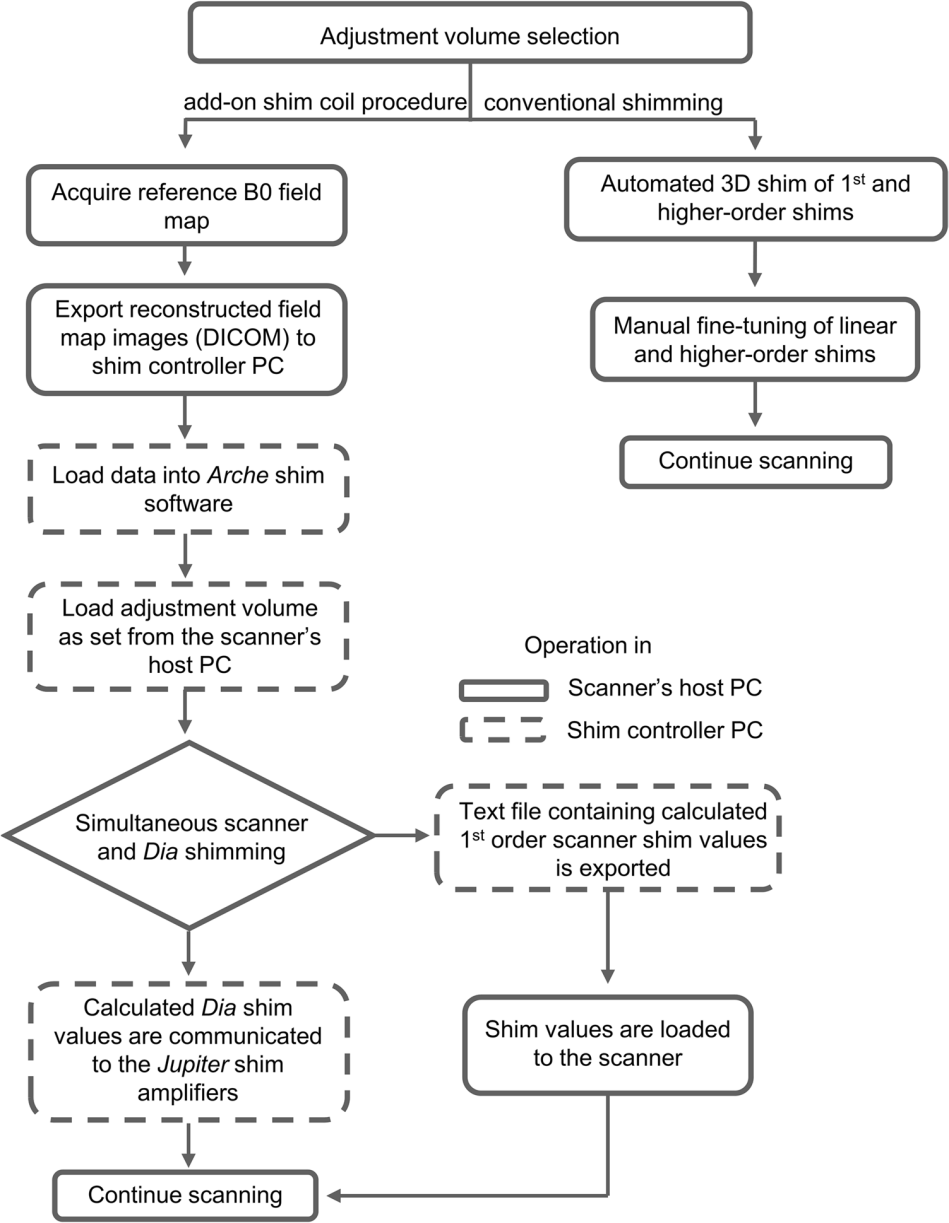
Flowchart providing an overview of the shimming process. Left: the add-on shim coil procedure Right: conventional shimming procedure

The shimming procedures using the 16-channel add-on local shim coil in combination with the scanner’s shimming system involved the steps outlined below and depicted in Fig. 3, to achieve a high 3D volumetric magnetic field homogeneity around the prostate.

For experimental shimming with the shim coil array, we identified a rectangular in vivo volume, indicated for B_0_ homogeneity optimization, known as the adjustment volume. This volume was precisely positioned on T2W localizer images, on the scanner’s host PC and software, selecting the prostate while minimizing the inclusion of surrounding tissues. Next, a double echo gradient echo pulse sequence was acquired for offline mapping of the B_0_ field within the adjustment volume. After obtaining the B_0_ field map, the optimal combination of 1st-order shim values of the MR scanner along with the settings for the add-on shim coil array was calculated on the shim controller PC. The higher-order shim values of the MR scanner were left unchanged at the tune-up values. To minimize B_0_ field disturbances within the adjustment volume, we used a least-squares minimization approach of the sum of the original B_0_ field heterogeneity and the targeted combined B_0_ shim field of the 1st-order scanner shims and the add-on shim coil array. The shim values were computed using a non-iterative approach via the *Arche* shim software, which employed a constrained convex optimization algorithm. Once calculated, these shim values were applied to the scanner’s 1st order shims in the interactive Adjustments window, and to the corresponding shim array amplifiers and coils on the separate shim controller PC.

As a reference standard, we used the MR scanner's 3D map shimming routine to calculate all available shim values (1st and 2nd order), specifically optimized for the prostate, following the protocol used in clinical routine. This was complemented by manual fine-tuning, where an experienced spectroscopist made interactive adjustments to the scanner settings. Our consistent goal during manual fine-tuning was to achieve a magnitude full width at half maximum (FWHM) of approximately 30 Hz within the adjustment volume around the prostate. In many instances, we achieved better results, well below the 30 Hz target.

The MR scanner's 3D map shimming routine settings were adjusted using the scanner’s clinical “fine-tune” routine, which minimizes the FWHM of the water resonance in the prostate. In contrast, add-on local shim coil array optimization minimized the ΔB₀ standard deviation within the prostate VOI. While the two methods use different metrics, this reflects the available control for each system.

#### Imaging protocol

The anterior modules of the add-on shim coil were fixed to the RF body receive array coil, while the posterior modules were fixed under the mattress of the patient bed. During subject positioning, care was taken to ensure the optimal placement of the prostate between the anterior and posterior shim coils.

The MR acquisition protocol and associated settings are detailed in Table 1. The acquisition procedure consisted of localizer scans, B_0_ field maps, T_2_-weighted (T2W) imaging, DWI, and ^1^H-MRSI. B_0_ field maps were acquired with a dual-echo gradient echo sequence. The anatomical T2W scans were obtained in three orthogonal orientations using a clinical turbo spin-echo (TSE) pulse sequence. For DWI, we employed a spin-echo EPI sequence with a b-value of 50 s/mm^2^ and repeated the sequence with b-values 50, 400, and 800 s/mm^2^, from which an image corresponding to a b-value of 1400 s/mm^2^ was calculated.

**Table 1:** The MR acquisition protocol and associated settings

Sequence	B_0_	T_2_W tra	T_2_W sag	T_2_W cor	DWI		^1^H-MRSI
	dual-GRE	TSE	TSE	TSE	EPI—b50	EPI for ADC	GOIA sLASER
TR (ms)	8	5660	6000	6000	3200	3600	750
TE (ms)	2.46/4.92	92	96	96	63	63	88
FOV (mm)	410 × 410	256 × 240	400 × 400	350 × 350	256 × 240	256 × 200	77 × 56x56
Resolution (mm)	3.2 × 3.2x3.0	0.3 × 0.3 × 3.0	1.3 × 1.3 × 3.5	1.1 × 1.1 × 3.5	2.0 × 2.0x3.0	2.0 × 2.0x3.0	Nominal, 7 × 7 × 7 mm; true spherical volume, 1.0 cm^3^
Averages	1	2	2	2	3		6
Slice thickness (mm)	3	3	3	3	3	3	
TA (min)	1:33	2:10	1:20	1:20	1:38	4:50	4:38

*MR* magnetic resonance, *DWI* diffusion-weighted imaging, *MRSI* magnetic resonance spectroscopic imaging, *T*_*2*_*W* T_2_-weighted imaging, *tra* transverse, *sag* sagittal, *cor* coronal, GRE Gradient echo, *TSE* turbo spin-echo, *EPI* echo-planar imaging, *GOIA* gradient offset independent adiabaticity, *sLASER* semi‐adiabatic localization by adiabatic selective refocusing, TR repetition time, *TE* echo time, *TA* acquisition time,

The 3D ^1^H-MRSI acquisition, a semi-LASER sequence with gradient offset independent adiabaticity (GOIA) pulses was used [34]. A slice-selective Shinnar-Le Roux optimized 90° excitation pulse (duration 4 ms) was followed by two pairs of refocusing pulses. The GOIA refocusing pulses with WURST(n,m) modulation had a duration of 8 ms, a bandwidth of 3 kHz, a maximum B₁ amplitude of approximately 718 Hz, and a gradient modulation factor of f = 0.9. The echo time (TE) was set to 88 ms. Lipid and water signal suppression was achieved using MEGA editing pulses placed after the first GOIA refocusing pulse. The MEGA pulses had a bandwidth of 1.6 ppm, a duration of 12.8 ms, and an RF amplitude of 260 Hz. Outer volume suppression bands were placed around the prostate volume to minimize lipid contamination. The acquisition matrix was an elliptically sampled k-space matrix of 8 × 8 × 11 phase encode steps zero filled to 16 × 16 × 16 after hamming filtering.

To minimize effects of motion between separate parts of the examination and to compare the two shimming approaches (add-on coil array vs reference standard), the shimming procedure was repeated twice during the protocol, involving separate B_0_ field acquisitions and recalculating shim values before both the DWI acquisition and the ^1^H-MRSI pulse sequence in both separate shimming approaches.

### Shim coils calibration

Reference B_0_ field maps from each individual shim coil were acquired on a phantom and served as the reference calibration data set. The positions of the shim modules with respect to the system’s isocenter and the subject’s prostate during each pursuing scan session were detectable on T2W localizer images by the fiducial markers embedded in the housing. As these shim modules vary in each subject and table position from the initial phantom shim coil calibration session, detection of fiducial markers on T2W localizer images for each subject is crucial. This was done manually by the user in the shim software (MR Shim, Germany) graphical user interface (GUI). Using these newly identified fiducial marker locations, rigid transformations, encompassing translation and rotation, were automatically calculated utilizing singular-value decomposition[35], and the magnetic field generated by each shim coil was calibrated to match the current position and orientation of the markers in each subject[36]. The total overhead calculation time when using the add-on shim system (including data transfer, fiducial marker detection and shim calculation) was approximately 2 min.

### Initial proof of concept

In this study a total of 16 local shim coils and the scanner-provided 1st and 2nd order spherical harmonic shim coils were available. Theoretically, the higher the number of employed shim coils, the more uniform the achievable shimmed field. In the initial stages of this work, we indeed used all available shim coils (add-on + 1st + 2nd order spherical harmonics) for shimming, illustrating a successful proof-of-concept implementation of the add-on shim array (Supplementary Fig. 2, vol A). However, very variable results were acquired, with cases in which the measured shimmed magnetic field map deviated substantially from the predicted maps (Supplementary Fig. 2, vol B: the measured field map after shimming deviated from the predicted value by more than 7 Hz).

Suspecting a disadvantageous interaction between possibly imperfect higher-order spherical harmonic fields, bowel motion and our add-on shim array we hypothesized that the 2nd order shims, while theoretically improving field homogeneity in the VOI, may reduce robustness against bowel motion. The magnetic fields generated by higher-order spherical harmonic coils are known to rapidly drop off further from the iso-center due to their geometry. This rapid drop-off contributes to a very inhomogeneous field outside the volume of interest (VOI) when shimming smaller regions of interest. This not only increases the chance of failure of global lipid/water suppression pulses and/or contributes to spurious signal stemming from outside the VOI in spectroscopy studies [37], but will additionally make the shim quality very susceptible to perturbations caused by motion.

Therefore, we simulated the robustness of two different shim strategies against motion, as described in Sect. "Initial proof of concept" and concluded that using the add-on shim coils in combination with only 1st order spherical harmonics shims of the scanner was the preferred way to go.

### Shim strategy for robust and motion-insensitive shimming

A simulation study was conducted to assess the robustness of two different shim strategies:1) using the add-on shim coils in combination with only 1st order spherical harmonics (add-on + 1st), 2) using the add-on shim coils in combination with both 1st and 2nd order spherical harmonics (add-on + 2nd).

**Fig. 4:**
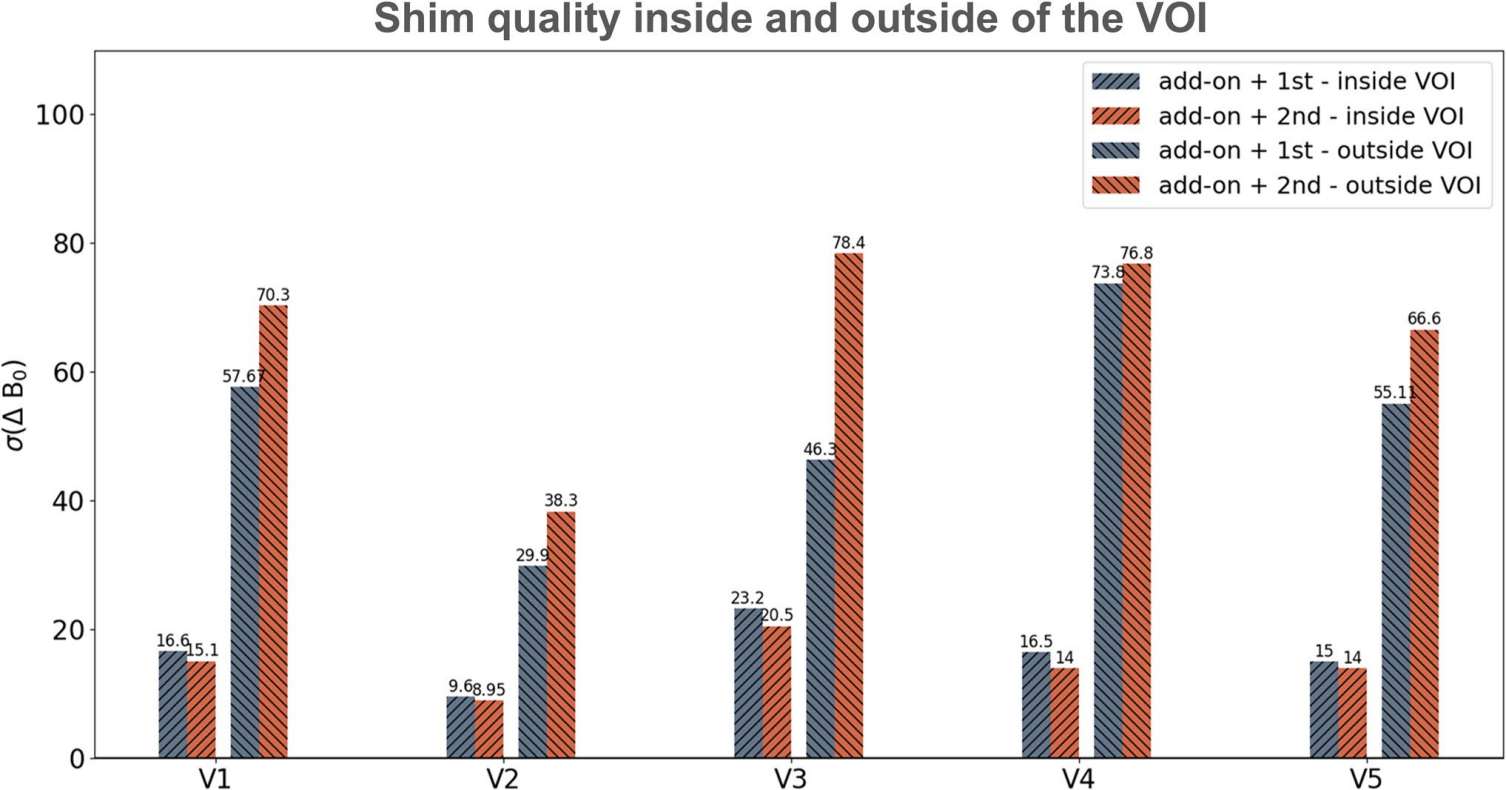
Comparison of simulated shim quality. For each volunteer, the standard deviation of the frequency offsets in the simulated shimmed map (both inside the prostate region and in an immediate ± 2 cm volume outside the prostate) are shown for two different shim configurations

Reference B_0_ maps from 5 healthy volunteers were used for the simulation study. First, the theoretically best attainable shim quality in the prostate region for each shim configuration was calculated. The resulting field homogeneity (as measured by the standard deviation of the frequency offsets) was compared between the two shim configurations both inside the prostate and in the immediate ± 2 cm outside the prostate in each direction. Figure 4 illustrates the bar plots of the calculated values for each setting. The shim optimization aimed to minimize the standard deviation of ΔB₀ within the prostate VOI using a least-squares approach.

**Fig. 5:**
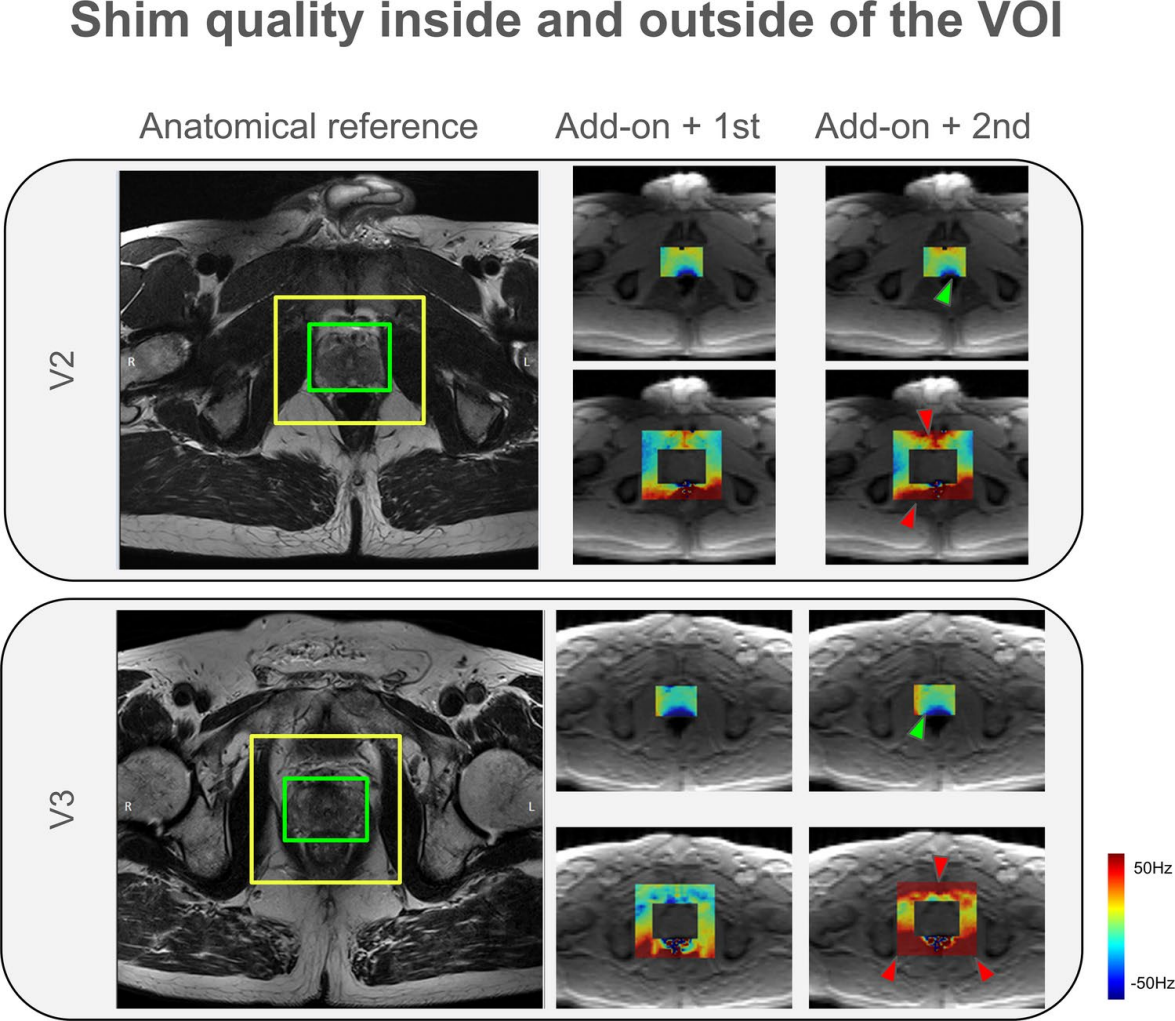
Plots of simulated shimmed maps inside and outside the volume of interest of the prostate. Simulated shimmed B0 maps overlaid on anatomical reference images are shown for two representative volunteers. The addition of 2nd order shims from the scanner introduced larger field deviations immediately outside the VOI (outside green box, within yellow box). The shimmed maps are shown for two different shimming configurations (add-on + 1st vs. add-on + 2nd). For each case, the maps of both inside (green box) and outside** (**yellow box) of the prostate region are shown. Green arrows point to areas where the inclusion of 2^nd^-order shims have resulted in an improvement inside the VOI. Red arrows point to areas with extreme and rapid field drop-off of field homogeneity outside the VOI

Τhe second shim configuration (add-on + 2nd order spherical harmonics) results in a better shim quality inside the prostate region (on average 1.67 Hz better than the add-on + 1st shim configuration across all volunteers). However, invariably, this advantage inside the VOI comes at the price of a worse field homogeneity in the immediate region outside the prostate (on average 13.5 Hz worse than the add-on + 1st shim configuration across all volunteers). As an example, the resulting shimmed field maps inside and outside the prostate region are shown in Fig. 5 for two representative volunteers.

Next, the robustness of each shim configuration against motion was assessed. A range of ± 1 to ± 9 mm displacement in each three directions (anterior–posterior, right-left, and head-foot) was considered. For each displacement, it was assumed that the underlying magnetic field is displaced by motion, but the prescribed imaging volume stays the same, as no prospective motion correction is employed. The effect of the motion on the shim quality of the original prostate position was calculated and compared for the two shim configurations. Additionally, another scenario where the anterior local shim coils (the only movable part of the shim system) also moves with the patient was considered for each case.

**Fig. 6:**
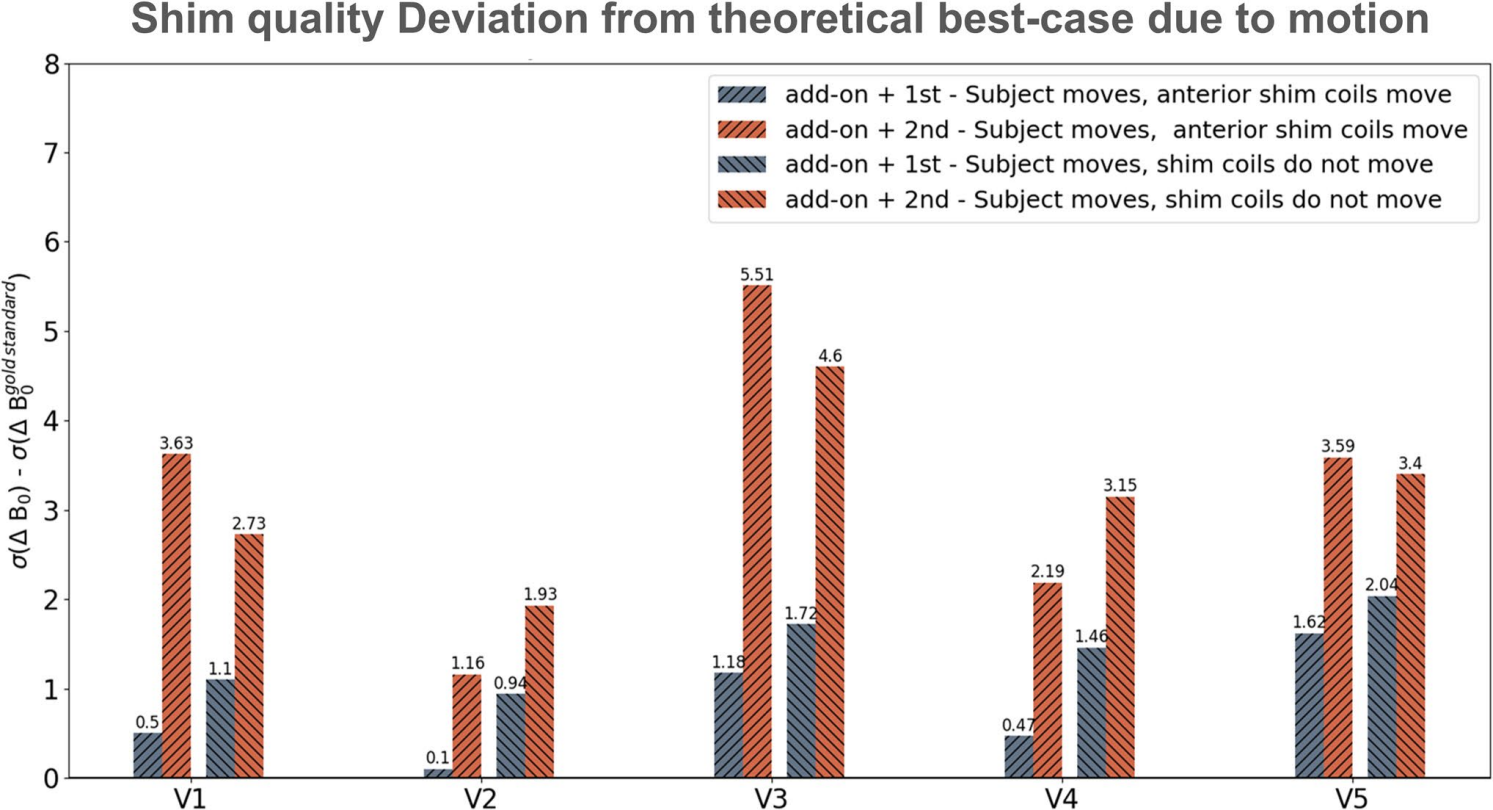
Susceptibility of shim quality to perturbations caused by motion. For each volunteer, the bar plots show the resulting aggregated shim quality for each shim configuration simulated over a range of motion (± 1 mm to ± 9 mm) in each three directions (AP,RL,HF). An additional scenario where the anterior shim coils also move are also shown. Each bar represents the deviation of the shim quality from the reference shim

Figure 6 shows the bar plots of the aggregated results showcasing how much the resulting field homogeneity deviates from the “reference” shim in each case. The reference shim is the best achievable shim using all available shim coils in a no-motion scenario (i.e. add-on + 2nd). It is important to study this metric since the goal here is to challenge the assumption that the configuration resulting in the reference shim in a no-motion scenario should be used without question. The goal is to understand how quickly each shim configuration strays from this reference as a result of even small amounts of motion. The results confirm that in every case the add-on + 1st shim configuration stays much closer to the reference shim. However, the add-on + 2nd order shim quickly loses its original advantage and results in a much worse shim, with an average deviation of 3.2 Hz across all volunteers and motion cases (compared to 1.1 Hz for the add-on + 1st scenario).

In light of this simulation study, the add-on + 1st shim configuration was chosen as the method of choice for the test of this study, as it resulted in a more stable shim and a more homogeneous field in the immediate region outside the VOI.

### Influence of add-on shim coil array on MR system performance

Tests were conducted using the homogeneous Siemens spherical D240 body loader tissue phantom to ensure compliance with performance criteria outlined by the National Electrical Manufacturers Association (NEMA) and the Acceptance Testing and Quality Assurance Procedures for Magnetic Resonance Imaging Facilities (AAPM). These tests aimed to evaluate RF interactions resulting from the presence of the shim coils. Measurements were conducted using body and spine array coils for signal reception and were performed twice: once with the shim coils present but without current running through them, and once without the shim coils.

Flip angle maps were obtained using the turbo flash B1 + mapping product sequence (tfl_b1map) with the following parameters: TR/TE 5280/1.8 ms, field of view (FOV) 300 × 300 mm^2^, base resolution 64 × 64.

Additionally, a 2D multi-slice T2-weighted (T_2_W) spin echo pulse sequence was employed with the following parameters: TR/TE 6000/96 ms, field of view (FOV) 350 × 350 mm^2^, 70 slices of 3.5 mm, and base resolution 256 × 256. From these images, the following parameters were assessed:

1. the signal to noise ratio (SNR), were a noise region and signal (S) region (Supplementary Fig. 3) where selected on the slice on iso-center and SNR was calculated according to

1 $${SNR}_{NEMA}=\frac{\sqrt{2\overline{\mathrm{S}}}}{\sigma }$$

where σ is the standard deviation of the noise in the noise region.

2. percentage of signal ghosting, where a signal region (*S*), and two sets of background regions (one in the frequency-encoding direction (*S*_*FE1*_, *S*_*FE2*_) and one in phase-encoding direction (*S*_*PE1*_, *S*_*PE2*_)) (Supplementary Fig. 4) were selected on the slice at iso-center and the percentage ghosting signal was calculated, through the ghosting ratio (*GR*), according to.

2 $$GR=100\left|\frac{\left(\overline{{S }_{FE1 }}+ \overline{{S }_{FE2}} \right)- \left(\overline{{S }_{PE1}}+\overline{{S }_{PE2}} \right)}{2\overline{S} }\right|$$

### Analysis

The analysis of the B_0_ and DWI data was conducted using Matlab (version 9.11 R2021b; MathWorks, Natick, MA).

#### B_0_-maps

To quantitatively assess B_0_ homogeneity, the standard deviation (SD) of off-resonance frequencies (ΔB_0_) was calculated per slice of the B_0_ maps within the Adjustment Volume.

The local *ΔΒ*_*0*_ variation was computed from the phase difference of the two acquisitions within a double gradient echo sequence with the parameters given in Table 1 [1]. The SD of the *ΔΒ*_*0*_ values was computed over the rectangular adjustment volume used for the second-order shimming, both on a slice-by-slice basis for each volunteer and over the entire volunteer cohort.

#### DWI analysis

The two image series with b-value of 50 s/mm^2^ (b50-maps) acquired with the add-on shim coil and with conventional shimming were by rigid registration matched to the transversal T2W scan as an undistorted anatomical reference, with interpolation of the EPI images to ensure resolution matching. A single reader manually segmented the entire prostate gland on each slice across the T2W series and the two b50-maps of both shimming methods. The contours of the b50-maps were aligned to the T2W images by matching the centroids of the corresponding contours.

Subsequently, the Dice similarity coefficient (DSC) [38, 39] was calculated to assess the agreement of the outlined prostate between both b50-images with the anatomical T2W images. In the DSC metric, the closer a value is to 1, the more precise the geometry of the prostate aligns in the b-50 DWI images as well as in the T2W images. This analysis was conducted on a slice-by-slice basis for each volunteer and for the entire cohort of volunteers. Statistical significance of the DSC’s was determined by a paired analysis using the Wilcoxon signed-rank test.

#### ^1^H-MRSI analysis

Spectroscopic imaging data were analyzed by quantifying the citrate (Cit) peak, a metabolite abundant in the prostate, and measuring its linewidth as an indicator of spectral quality. Fitting was performed using AMARES within the jMRUI software [40]. Although Cit consists of a strongly coupled spin system with a pulse sequence timing and B0 field-dependent complex line shape, we could model the center two lines of this shape with two coupled Lorentzian line shape models. This way, the possible presence of individual variation in the small satellite signals around the two central lines does not interfere with the linewidth assessment. In the fitting procedure, all spectra within the volume of interest (VOI) were included, followed by a quality control (QC) step. Spectral fits with a Cramer Rao Lower Bound (CRLB) of the linewidth smaller than 20% were accepted as good estimates of the linewidth. The percentage of the spectra passing the QC in both shimming techniques was calculated. Spectra that satisfied the QC criterion in both shimming protocols were retained for subsequent analysis steps. To assess the statistical differences in Cit linewidth between the two shimming techniques, a paired t-test for statistical evaluation was performed, both on an individual volunteer basis and for the entire volunteer cohort. To assess the overall quality of the MRSI datasets, a scoring system based on the expert consensus paper [4] was created. The following guidelines on FWHM of spectroscopy in the prostate at 3 T are established in that paper:

- 5 < mean FWHM < 8: Excellent
- 8 < mean FWHM < 11: Adequate
- 11 < mean FWHM < 14: Acceptable

- 14 < mean FWHM: Unacceptable

Based on this guideline a scoring system is devised, which takes into account not only the mean but the standard deviation (spread) of the FWHM values distributed across the prostate:

3 $$Score=100- \frac{\left(FWHM-6.5\right)}{8}*80-\left(\sigma FWHM-1.5\right)*10$$

The scoring system has a linear penalty for deviation of the average FWHM from 6.5 Hz (mean of excellent score according to the consensus paper). Additionally, there is a term penalizing the high spread of FWHM values in the VOI (> 1.5 Hz which is the acceptable spread in each of the consensus system tiers). A wider distribution of FWHM values indicates heterogeneity in the data and reduced reliability of the overall spectroscopic information. The scoring formula reflects the expert guidelines by assigning a high score (100) for either a FWHM distribution of 8 ± 0 Hz or 6.5 ± 1.5 Hz.

The scores for each MRSI dataset were calculated and compared. Additionally, exemplary Cit linewidth metabolite maps for three slices from a single volunteer were generated, and histograms were created to visually depict the distribution of Cit linewidths among the volunteers.

## Results

The phantom results are presented in Table 2. The RF transmit efficiency was reduced by approximately 2% as a result of the presence of the local shim coils, confirming that all the necessary RF pulses and flip angles of the MRI imaging sequence were reasonably achieved. Additionally, no ghosting artifact was observed. The RF receive performance (SNR) was reduced by approximately 20%, which is not negligible. Representative B1⁺ maps (flip angle maps) and SNR maps acquired in the phantom, with and without the shim array present, are shown in Supplementary Figs. 5 and 6, respectively. This topic is addressed in more detail in the Discussions section. For the purposes of the current work, the presence of the shim coils did not affect the results of the comparison study, as the shim coils were always present for both cases of the comparison study, therefore the SNR baseline remained the same for both shimming cases.

**Table 2:** Phantom results

Metric	Presence of shim coils	Absence of shim coils	% difference with and without shim coils	Performance criteria
Average flip angles [degrees]	78.38	80.21	− 2.28	
SNR	863.87	1079.60	− 19.98	> 215
Ghosting [%]	0.33	0.34	+ 0.01	≤ 1%

All volunteers completed the full protocol successfully, except for one subject, V5, where an MRI system error prevented the DWI acquisition process.

**Fig. 7:**
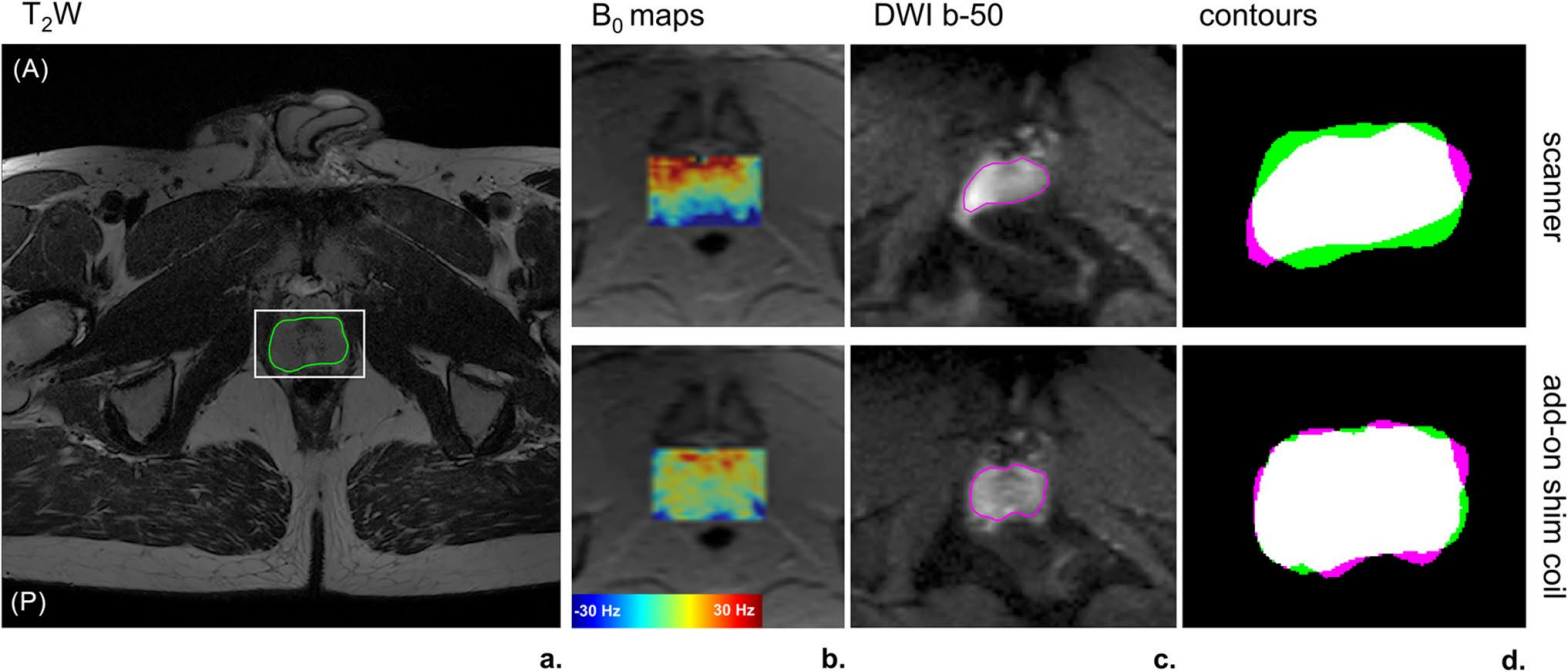
DWI analysis of an example slice in a subject (V3) using both conventional shimming (top) and the add-on shim coil (bottom) approaches** a** Transversal T2W image as an anatomical reference with the prostate outlined in green and the selected shimming adjustment volume (white frame) **b** B_0_ maps **c** b-50 EPI images with the prostate outlined in purple **d** b-1400 EPI images **e** resulting overlaid prostate segmentations of T2W contour (green) and b-50 DWI contour (purple) (scanner DSC = 0.87; add-on shim coil DSC = 0.94)

Figure 7 depicts the impact of the add-on shim coil on B_0_ maps and b50-maps derived from DWI, illustrated by an example case (V3). Specifically, for one slice the anatomical T2W reference is shown with the delineated prostate within the marked adjustment volume (white frame) (Fig. 7a). The B_0_ maps from both conventional shimming and the add-on shim coil highlighted a reduced frequency offset when using the add-on shim coil (Fig. 7b). The DWI b50-maps for both shimming techniques are depicted with their respective prostate contours (Fig. 7c). Overlaying the T2W image contour onto the b50-maps revealed improved alignment with the use of the add-on shim coil compared to conventional shimming (Fig. 7d), signifying less distortion in the b50-maps when employing the add-on shim coil.

**Fig. 8:**
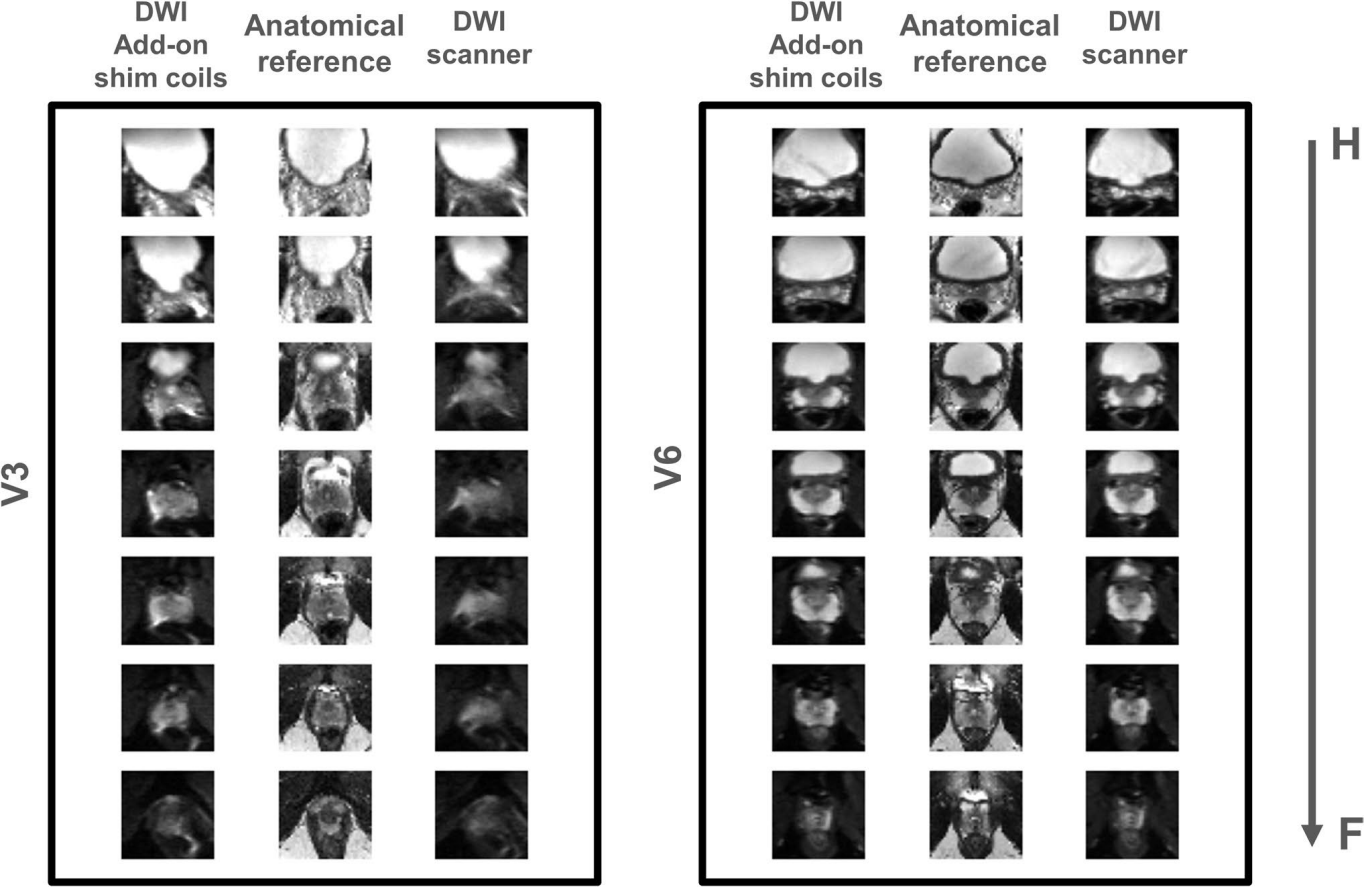
DWI image comparisons- DWI images across the prostate are shown for two volunteers. The anatomical references are shown in the middle column for each case. The DWI images of volunteer 3 showcase improvement in border delineation and geometric distortion when add-on shim coils are used. In the case of vol 6, excellent DWI images were obtained for both cases

The SD of ΔB0, extracted from B0 maps, and DSC, calculated per slice, were compared between both shimming techniques, on the entire cohort and on a per-volunteer basis. Using the add-on shim coil resulted in a statistically significant overall improvement in the shim quality (by 2 Hz) and in reducing distortion in b50-maps (Table 3). The statistical analysis on a per-volunteer basis revealed that in half the cases (3 out of 6 volunteers) statistically significant differences between the two shimming methods were observed. In two of these cases (volunteers 1 and 3), the add-on shim coil outperformed conventional shimming, resulting in a higher mean DSC, translating to less distortion. However, in one case (volunteer 6) shimming with the add-on device appeared to perform slightly worse than the conventional shim, although the effect size was very small. Examination of the raw data (Fig. 8) confirmed that albeit statistically significant, in this case the very small effect size meant that the two sets of images were already exhibiting near-perfect similarity to the anatomical reference. In contrast, Fig. 8 also shows that in the other statistically significant cases the add-on shims resulted in significantly better images. In volunteers 2, 4, and 7, no statistical difference was found between the DSCs of the two shimming methods. Indeed, in most of these cases the DSC values were consistently high, indicating high DWI image quality for both shimming cases with little room for further improvement. Out of all the volunteers, only one case (volunteer 7) showed a poor DSC coefficient for both shimming cases, despite achieving very good (< 15 Hz) shim quality for both approaches. Upon further examination of the raw data (Supplementary Fig. 7), this could be attributed to motion in HF direction between the anatomical T2W and DWI scans, introducing a mismatch between the anatomical reference and DWI geometries, hence rendering the similarity statistics inapplicable for this particular case.

**Table 3:** Overview of results on B_0_ map and DWI for all volunteers for conventional shimming and the add-on shim coil

Volunteer #	B_0_ for DWI		DSC from DWI		
	SD of ΔB_0_ *Hz*		DSC_mean_ ± SD		*p*-value
	Add-on shim coil	Conventional	Add-on shim coil	Conventional	
1	21.7	23.9	0.91 ± 0.01	0.88 ± 0.02	0.05*
2	12.4	12.8	0.92 ± 0.03	0.91 ± 0.02	0.87
3	18.1	24.7	0.93 ± 0.01	0.84 ± 0.02	0.03*
4	19.2	18.5	0.92 ± 0.02	0.92 ± 0.02	0.58
5					
6	11.6	13.3	0.94 ± 0.02	0.95 ± 0.01	0.02*
7	15.7	15.6	0.88 ± 0.02	0.88 ± 0.01	0.19
ALL	16.5 ± 3.6	18.5 ± 4.7	0.92 ± 0.02	0.90 ± 0.02	0.01*

Values in bold indicate statistically significant differences (*p* < 0.05)

**Fig. 9:**
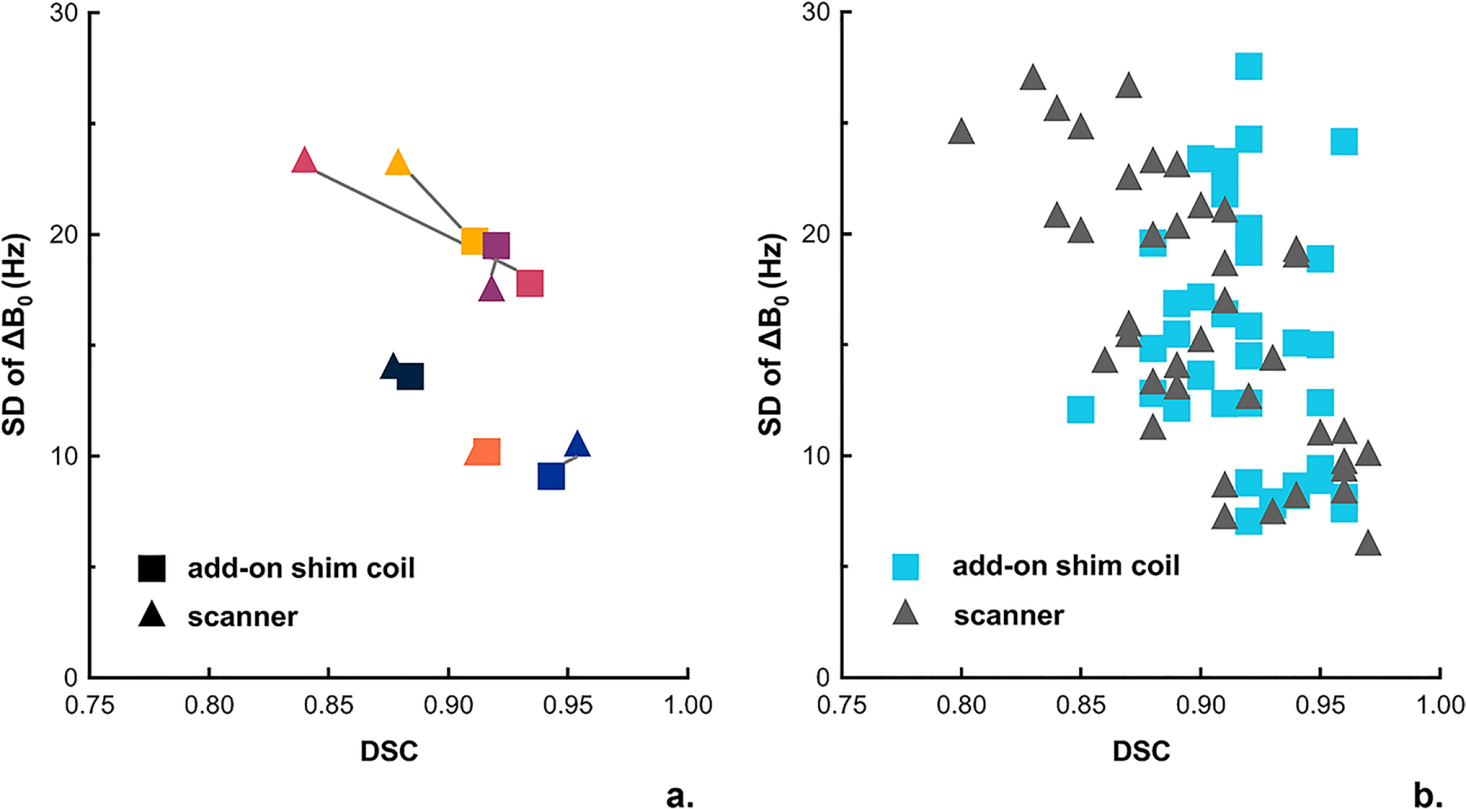
DWI analysis of the full cohort of examined subjects. **a** SD of ΔB_0_ as calculated from B_0_ maps in relation to DSC of the b-50 DWI when compared with T2W images, displayed on a per-volunteer basis **b** SD of ΔB_0_ calculated from B_0_ maps in relation to the DSC of the b-50 DWI when compared with T2W images, presented on a per-slice basis

Examining the potential relationship between the B_0_ inhomogeneity reflected by the SD of ΔB_0_ from the B_0_ maps, and the distortion of EPI images in b50-maps, the SD of ΔB_0_ of each volunteer was plotted in relation to the mean DSC (Fig. 9a). In volunteers with an SD of ΔB_0_ exceeding 20 Hz in conventional shimming (two out of the six cases), the add-on shim coil achieved a lower SD of ΔB_0_ and higher DSC values, indicating an improvement in magnetic field homogeneity. Additionally, we plotted the values of all slices within the prostates of all the volunteers (Fig. 9b). In the per-slice analysis, the slices with lower DSC values in conventional shimming displayed higher DSC values when using the add-on shim coil.

**Fig. 10:**
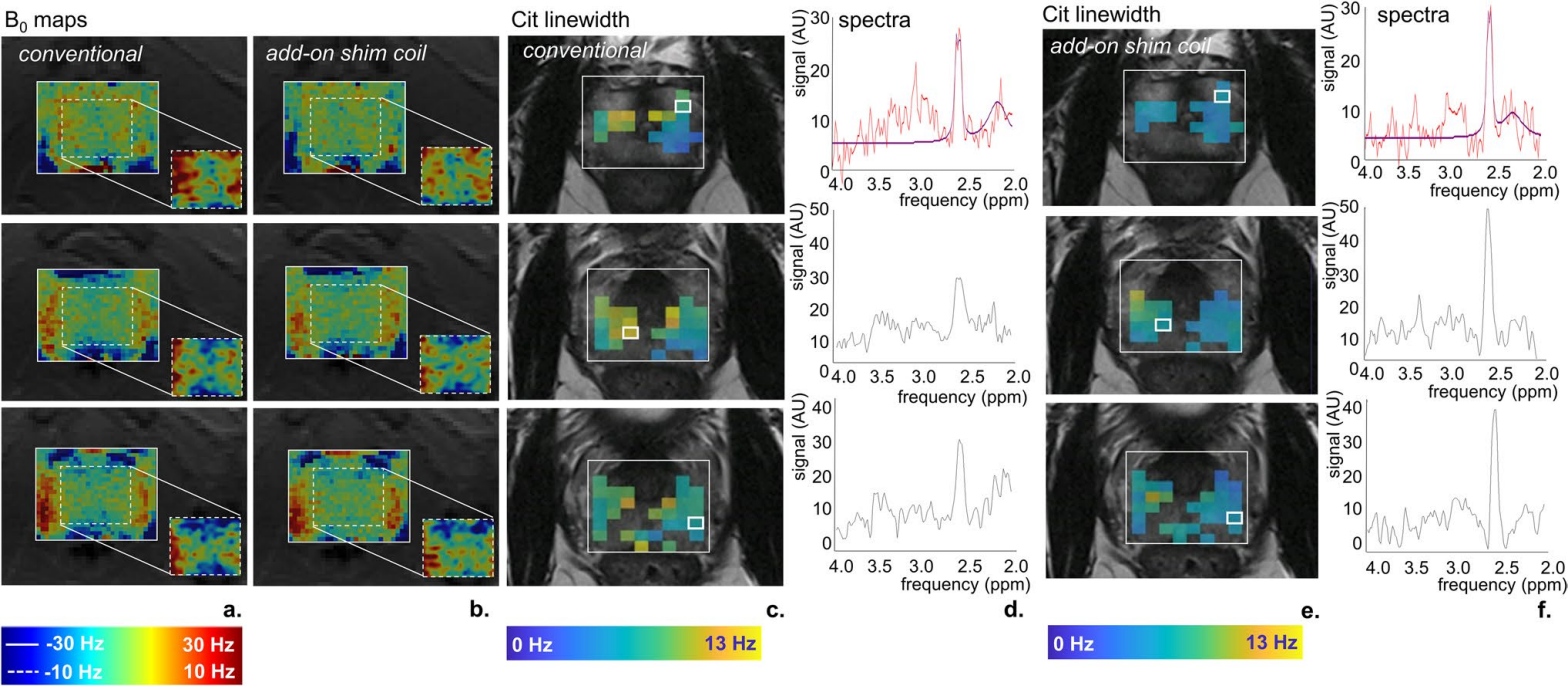
Shimming comparison in MRSI of conventional shimming and the add-on shim coil in three slices of an exemplary volunteer (V7). **a** B_0_ maps with conventional shimming **b** B_0_ maps with the add-on shim coil. The solid line frame represents the full adjustment volume with a frequency range of [− 30, 30] Hz, while the dashed line frame is zoomed in with a frequency range of [− 10, 10] Hz. **c** Cit linewidth maps from conventional shimming. The voxels that successfully passed the QC in both shimming approaches are visualized in the figure. **d** Spectra from indicated voxels in column c. In the top spectrum, the signal is in red and the fitting profile is in purple. **e.** Cit linewidth maps from the add-on shim coil. The voxels that successfully passed the QC in both shimming approaches are visualized in the figure. **f.** Spectra from indicated voxels in column e. In the top spectrum, the signal is in red and the fitting profile is in purple

Figure 10 displays the impact of both shim methods on ^1^H-MRSI, showing the comparison using B_0_ maps and Cit linewidth maps. Results from conventional shimming and the add-on shim coil are presented across three slices of an exemplary volunteer (V7). B_0_ maps offered insights into both the full adjustment volume and a zoomed-in region (Fig. 10 a and b), revealing reduced frequency offsets with the add-on shim coil, particularly evident in the zoomed-in panels. Citrate linewidths of voxels that successfully passed the QC in both shimming approaches narrowed with the add-on shim coil (Fig. 10 c and e), which is also visible in individual spectra (Fig. 10 d and f).

In terms of quantitative and statistical analysis, the QC evaluation revealed a comparable percentage of voxels within the complete VOI meeting the quality test criteria for both shimming techniques, achieving the same success rate of 43% for both the add-on shim coil and conventional shimming. The statistical analysis was performed on all spectra that successfully passed the quality assessment in both techniques, revealing a significant difference in Cit linewidths (*p* < 0.05) between the two approaches along the full cohort. Specifically, the mean Cit linewidth value for the add-on shim coil method was 7.9 ± 3.6 Hz, whereas conventional shimming resulted in a significantly higher value of 8.3 ± 5.7 Hz (*p* = 0.018). The calculated scores for both shimming configurations are also shown in the same table.

On a per-volunteer basis, in all cases the use of the add-on shim coils resulted in a lower variability (lower SD) of FWHMs, indicating a more consistent quality across the VOI. Looking at the calculated scores, the use of the add-on shims resulted in a higher score of the MRSI data quality in all cases. Based on the quality tiers defined according to the expert consensus metrics in the Methods section, the conventional shimming method resulted in two “Excellent” (vol 2, 6), one “Adequate” (vol. 7), one “Acceptable” (vol. 3) and three “Unacceptable” (vol. 1, 4,5) datasets. Using the add-on shim coils, the results consistently improved to four “Excellent” (vol.2, 3, 6, 7), one “Adequate” (vol. 4) and two “Acceptable” (vol. 1, 5) datasets.

**Fig. 11:**
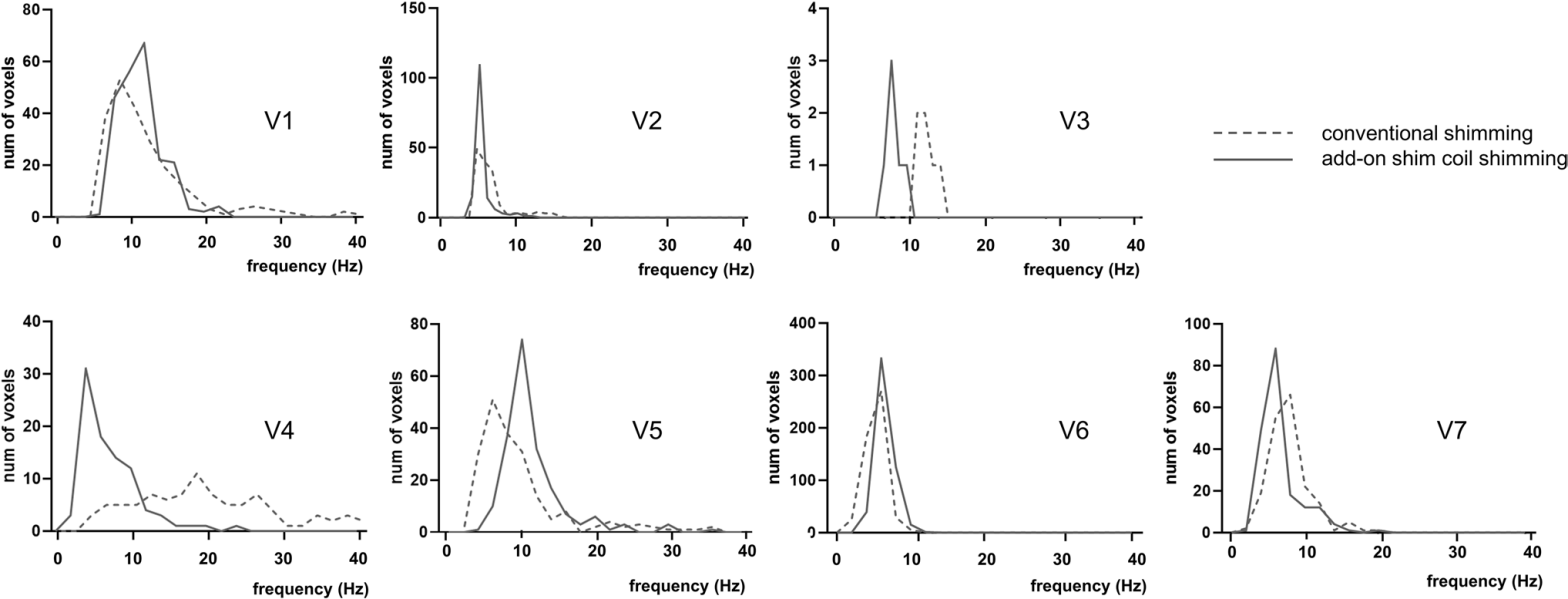
Cit linewidth distribution histograms on a per-subject basis. Cit linewidths from ^1^H-MRSI acquisitions with the add-on shim coil (solid line) and conventional shimming (dashed line) from all voxels that passed QC per volunteer. On average, the mean Cit linewidth improved from 8.3 Hz with conventional shimming to 7.9 Hz using the add-on shim coil (*p* = 0.018)

For a more detailed perspective on Cit linewidth results within each subject, Fig. 11 presents histograms depicting the distribution of Cit linewidths. The panels in this figure include all voxels that successfully passed the QC assessment in both shimming approaches. A global shift of the histogram towards smaller linewidths demonstrates an overall improvement in linewidths across the majority of volunteers. This improvement was especially evident in cases like V5, where conventional shimming showed particularly poor results.

## Discussion

Achieving a homogeneous B_0_ field in the organ of interest is a fundamental requirement for successful MR acquisitions. High-quality B_0_ shimming hinges on various crucial factors, such as carefully calibrating the B_0_ shim coil hardware of the MRI system, employing robust methodologies to evaluate the B_0_ homogeneity in the volume of interest, as well as precise computation of shim coil currents to modify the subject-specific field homogeneity with additional B_0_ shim coils[4]. In this study, motivated by the fact that specific strong localized field inhomogeneities, such as those caused by gas in the rectal cavity, can be more effectively addressed using targeted fields generated by a local shim array, our objective was to enhance B_0_ homogeneity in prostate imaging using an add-on shim coil array.

Achieving and maintaining a uniform magnetic field in the prostate is a challenging task, as this organ is surrounded by different tissue types and prone to considerable motion (in contrast to most local shim coil studies, which are conducted in the brain). Therefore, in the current work, a simulation study was performed to determine the best combination of local and global shim coils for achieving a robust shim. It was concluded that the combination of the local array of shim coils with only the 1st order global shims provided the most robust performance in the presence of motion, while also maintaining a gradual drop-off of field homogeneity in the immediate vicinity surrounding the prostate, reducing the chance of spurious and nuisance signals stemming from outside the organ.

We assessed the performance of the add-on shim coil array against the conventional shimming methodology of the MR scanner for prostate imaging (including higher-order shim coils), as typically performed in clinical practice, supplemented by manual fine-tuning. Manual fine-tuning is a common practice in prostate spectroscopy, aiming to achieve optimal field homogeneity within the constraints of the conventional scanner's approach. The effectiveness of the two shimming procedures was evaluated on B0 field maps, DWI, and ^1^H-MRSI acquisitions.

DWI, facilitated by EPI sequences, is crucial for accurate localization of prostate malignancies, especially in the peripheral zone of the prostate gland[41, 42]. Poor shimming can compromise the diagnostic value of mpMRI [43, 44], particularly in patients with metallic hip arthroplasties, where susceptibility artifacts are commonly seen [45]. Additional shimming can improve data quality, aiding in precise diagnoses of conditions like BPH, prostatitis, or cancer. To evaluate the add-on shim coil's effectiveness with DWI, we assessed image distortion using DSC on b50-maps with an EPI sequence due to its short acquisition time (TA = 1:38 min). As a reference, we used T2W images acquired through a TSE sequence, known for its resilience to B0 inhomogeneities [46] due to multiple refocusing pulses preventing the accumulation of phase errors [47].

^1^H-MRSI is a valuable tool for assessing tissue metabolites [48] and is substantially affected by B0 field inhomogeneity. The spectral quality of MRSI data is highly influenced by B0 field variations. Intra-voxel variations cause line broadening, and inter-voxel variations across the organ introduce line shifts from one voxel to another [37, 49]. Addressing these is crucial for accurate metabolic assessments, as spectral overlap can adversely affect the quantification of key metabolites.

The experimental results from B0 mapping performed twice on a cohort of seven subjects showed an overall improvement of B0 inhomogeneity using the local shim array across the entire cohort (Tables 3 and 4), confirming the advantage of the local shim array in addressing local B0 field inhomogeneities.

**Table 4:**
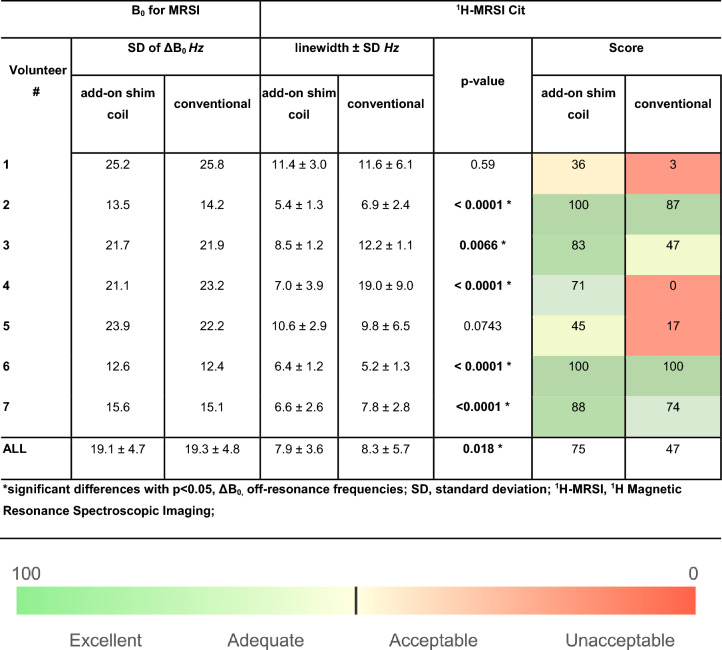
Overview of results on B_0_ maps and MRSI all volunteers for conventional shimming and the add-on shim coil

For DWI imaging, in approximately 30% of cases, the overall DWI image quality using conventional scanner shimming was poor (DSC < 0.9) showcasing distortion artifacts as a result of field inhomogeneity. In these cases, the addition of the local shim array effectively mitigated these artifacts and significantly improved the image quality.

When the results from both DWI and B0 maps were analyzed on a per-slice basis, it became evident that when the add-on shim coil was used, in certain slices a high SD of ΔB0 as well as high DSC values were observed (Fig. 9). This contradictory finding can be attributed to the following factors: 1) the SD of ΔB0 is calculated on the complete cuboid 3D adjustment volume around the prostate, while the DSC value specifically pertains to the prostate contour within this volume, 2) patient motion during the scan could have contributed to a lack of apparent correlation between the slice-by-slice B0 SD and DWI image quality. This further suggests that using a shim configuration that is robust against small perturbations (such as motion) is an important factor to consider when shimming the prostate.

Importantly, in one case of DWI imaging, the subject exhibited motion during the course of the DWI scan session, and upon inspection of the DWI data quality, the local shim coils still demonstrated reduced geometric distortion and signal pile-up artifacts compared to scanner shims, confirming the robustness of our approach against motion-induced fluctuations.

A similar trend was observed for MRSI. In around 40% of the cases, the mean FWHM of the Cit metabolite peak was unacceptably broad when using conventional scanner shimming (judged against the FWHM ranges set forth in the expert consensus). The local shim coils were able to significantly improve the data quality in all these cases, reducing the FWHM to the “Acceptable” and even “Adequate” range.

For the remaining cases, the DWI and MRSI datasets already exhibited a good data quality at baseline and although on a slice-by-slice and local level differences were observed, on a macro level the effect size was small and the data quality was preserved for both shimming cases. In cases where DSC was already high with conventional shimming, the add-on shim coil still holds potential for enabling higher resolution acquisitions or more distortion-prone sequences, where enhanced B0 homogeneity may become more critical.

It should be noted that the overall improvement in the linewidths in the ^1^H MRSI study across all volunteers when using the add-on shim coils was statistically significant despite the marginal improvement in the SD of ΔB0 (only around 0.2 Hz overall improvement). This is likely due to the longer scan times of the MRSI, which increases the chance of perturbations during the scan as a result of physiological as well voluntary motion. This further confirms the robustness of the shim method against perturbations, since the MRSI linewidths were still scored higher (average score of 75/100) for the local shims.

### Challenges and future aims

This study presents some challenges and limitations. Although the performance criteria set by NEMA and AAPM guidelines were satisfied, the presence of shim coils had an effect on the SNR of the sample or subject. Even though the effect on transmit efficiency was almost negligible (~ 2%), a 20% receive performance penalty was caused by the presence of shim coils as measured by the SNR of the T_2_W images. To maintain consistency, the shim coils were present for both of the comparison cases throughout this study so that any change in the image quality can be directly attributed to changes in B0 field homogeneity rather than potential confounding factors related to RF interaction. For future iterations, it is crucial to minimize the interaction of the local shim coils with the RF receive arrays by leveraging the recently published methods [50] to achieve “RF transparent” multi-turn shim coils.

The selection of the adjustment volume is important to ensure robust and repeatable results, especially in abdominal positions where proximity to the rectum can introduce air into the adjustment volume, potentially impacting shimming accuracy. Additionally, it is essential to confirm a stable water resonance frequency between scans and repeat the shimming process if a difference is detected to account for potential motion, organ displacement, and alignment issues between the organ of interest and the adjustment volume.

As a prospective goal, our next aim involves extending this approach to patient populations where the use of medication to suppress bowel motion is standard practice. This group often includes individuals with metallic hip implants that strongly deteriorate local field homogeneity. Enhanced shimming flexibility could offer substantial benefits to this group. Furthermore, concerning equipment development, investigating the incorporation of the shim coils into the RF receive array and automated detection of their position, but without mutual interaction between RF and shim array, as well as implementing a feedback control system to continuously monitor shimming quality between consecutive acquisitions is of interest. Such a system would enable prompt re-shimming if a decline in quality is detected. Additionally, the exploration of dynamic shimming techniques holds promise in further enhancing shimming performance within the targeted organ.

## Conclusion

In this work, we presented the first version of a local external add-on shim coil array, tailored for prostate applications. Extensive assessments involving robustness of the shim quality, effects on B_0_-maps, EPI-based DWI, and ^1^H-MRSI have provided evidence of the effectiveness of this shim coil array. Across seven volunteers, the use of the add-on shim array reduced the standard deviation of B0 inhomogeneity by ~ 2 Hz, improved the Dice similarity coefficient in DWI from 0.90 ± 0.02 to 0.92 ± 0.02 (*p* = 0.01), and decrease in citrate linewidths from 8.3 ± 5.7 Hz to 7.9 ± 3.6 Hz (*p* = 0.018), all compared to the reference standard of conventional 1st and 2nd-order shimming. Our primary objective was to show the feasibility of this approach and to enhance B_0_ field homogeneity within the prostate, leading to improved image quality and reduced image artifacts in DWI and MRSI, potentially enhancing precision in MR examinations of the prostate.

## Supplementary Information

Below is the link to the electronic supplementary material.

Supplementary file1 (DOCX 1310 KB)

## Data Availability

The data that support the findings of this study are available from the corresponding author upon reasonable request.
