# Supplementary material for: Improving magnetic field homogeneity in prostate MR imaging and spectroscopy using an add-on local external shim coil array
Source: MAGMA. 2025 Sep 11;39(1):137–55. doi: 10.1007/s10334-025-01290-y (PMC12901270; doi:10.1007/s10334-025-01290-y)
Supplement: Supplementary file 1 — Supplementary file1 (DOCX 1310 KB) [file 10334_2025_1290_MOESM1_ESM.docx]

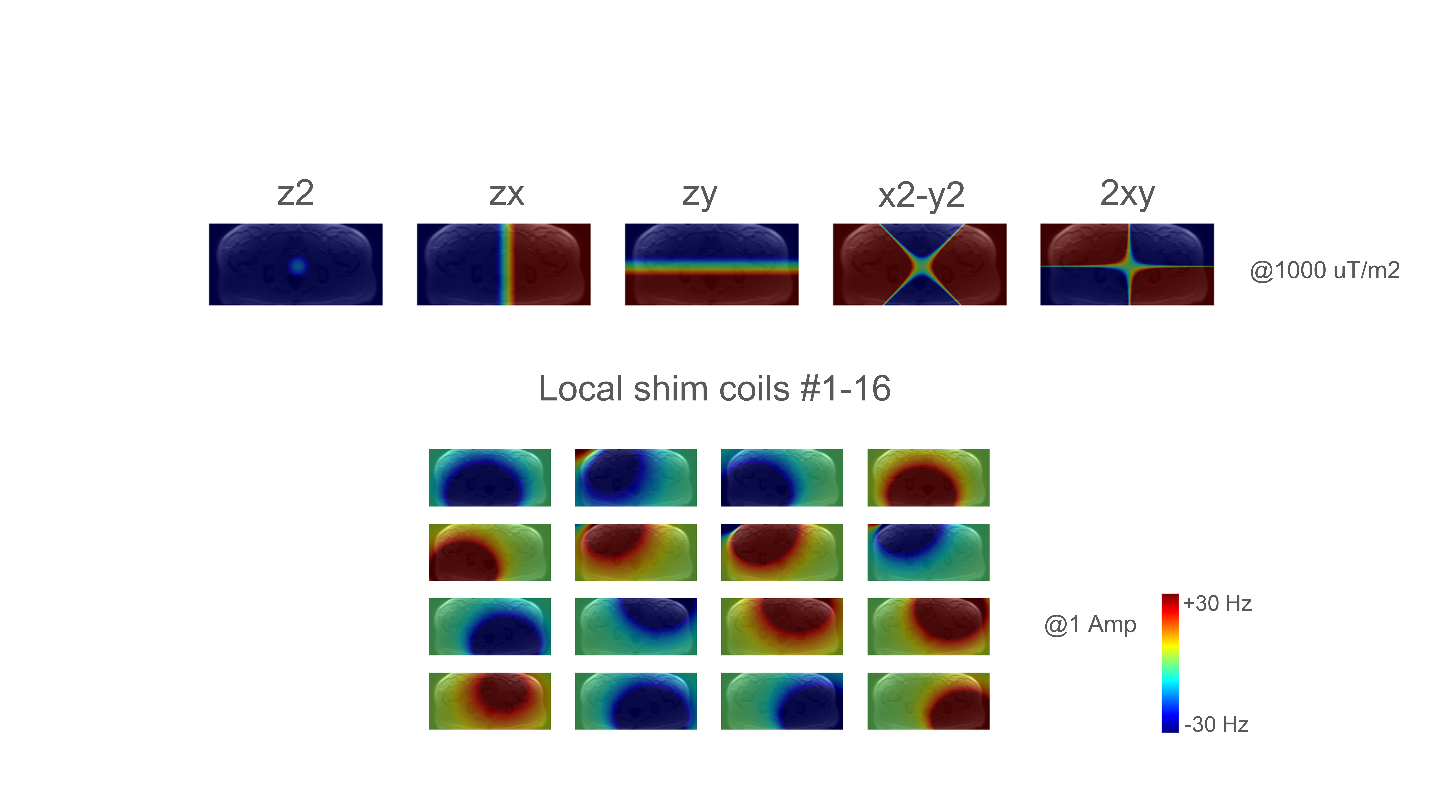


**Supp. Figure 1.** Example of generated field maps from scanner’s higher order shims and local shim coils shown on axial slice


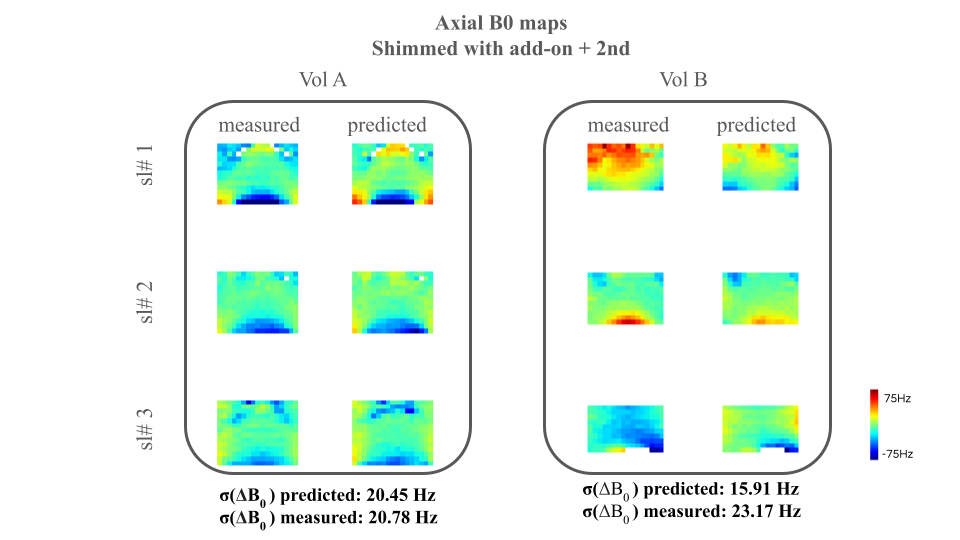


**Supp. Figure 2.** Predicted and measured B0 maps in 3 slices of the prostate of two representative volunteers from the initial phase of the project. The scanner’s 1^st^ and 2^nd^ order shims together with the add-on shim array were used . In the case of volunteer B (VolB) the measured B0 map was far worse (+ 7Hz) than the predicted map.


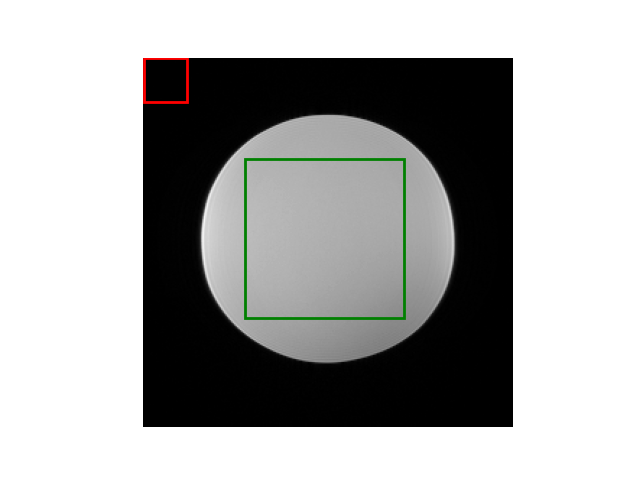


**Supp. Figure 3.** A noise region (red) and signal region (green) were selected on the slice on the iso-center and SNR was calculated according to the NEMA standard.


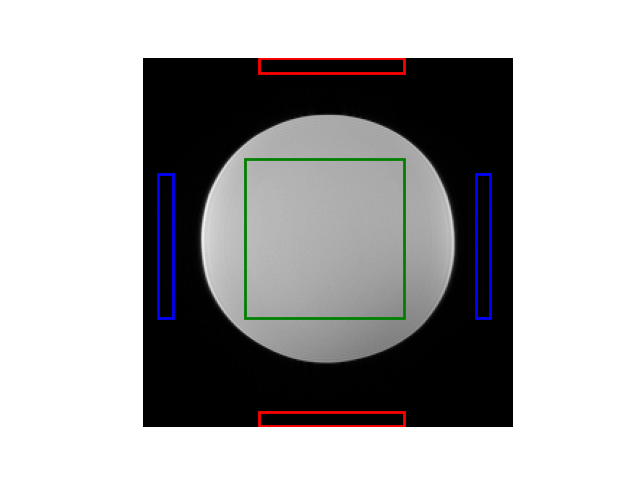


**Supp. Figure 4.** A signal region (S, green), and two sets of background regions one in the frequency-encoding direction ($S_{FE1,2}$,red) and one in phase-encoding direction ($S_{PE1,2}$blue) were selected on the slice on the iso-center and the percentage ghosting signal was calculated according to the AAPM report


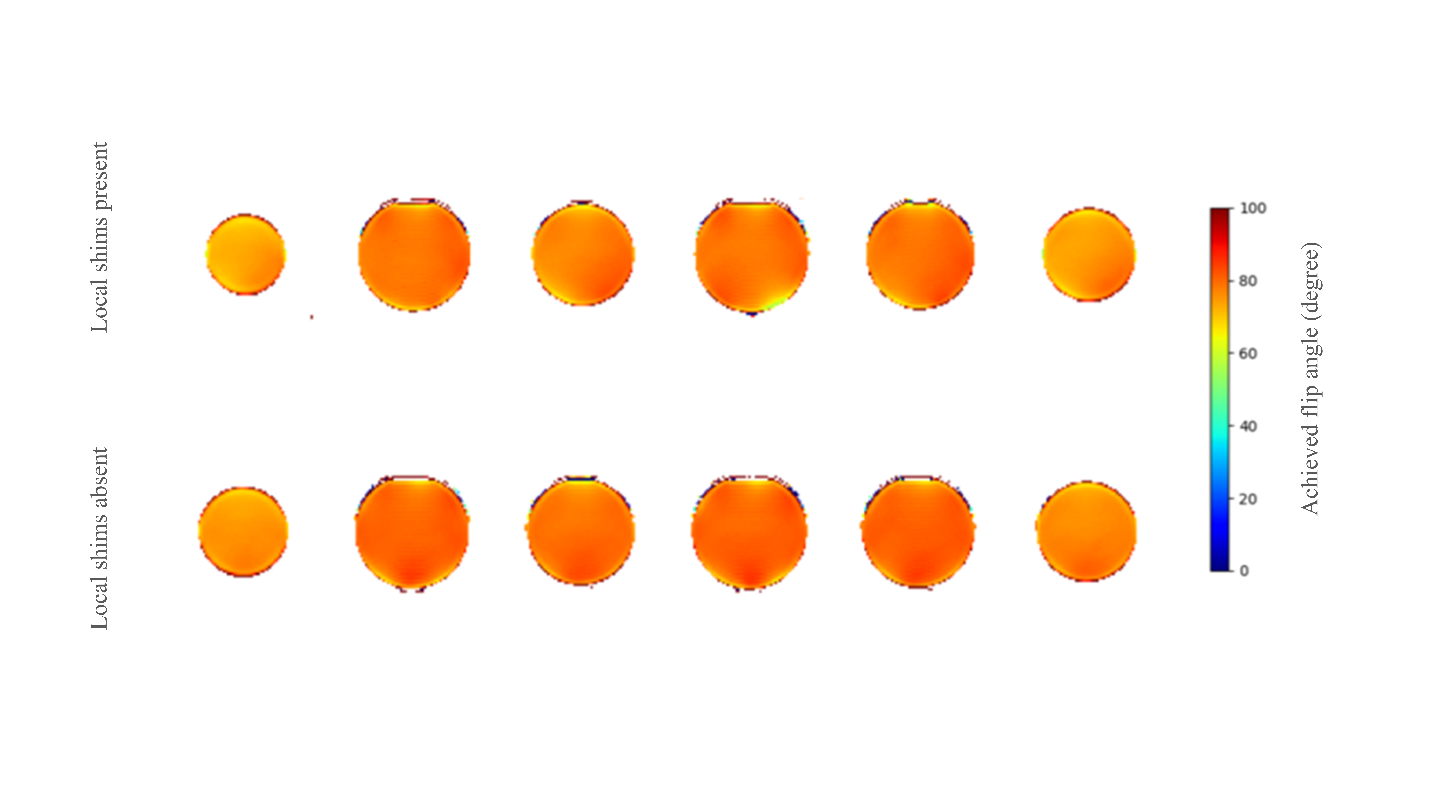
**Supp. Figure 5.** Flip angle maps: B1+ (flip angle) maps are shown in representative slices across the phantom. An average 2% difference in transmit efficiency was observed


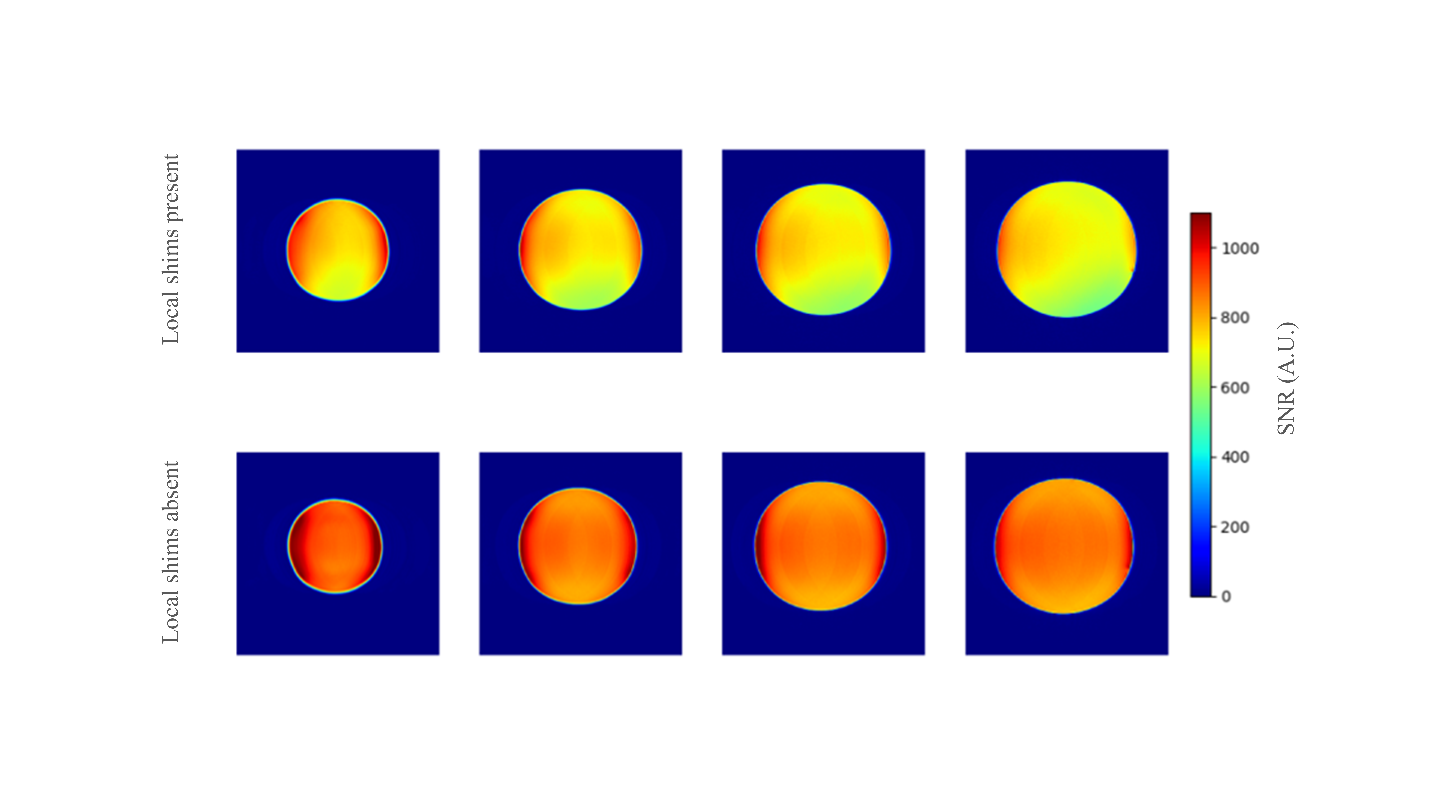


**Supp. Figure 6.** SNR maps shown in representative slices across the phantom. An average 20% difference in SNR was observed. For future iterations, it is crucial to minimize the interaction of the local shim coils with the RF receive arrays by leveraging the recently published methods[50] to achieve “RF transparent” multi-turn shim coils.


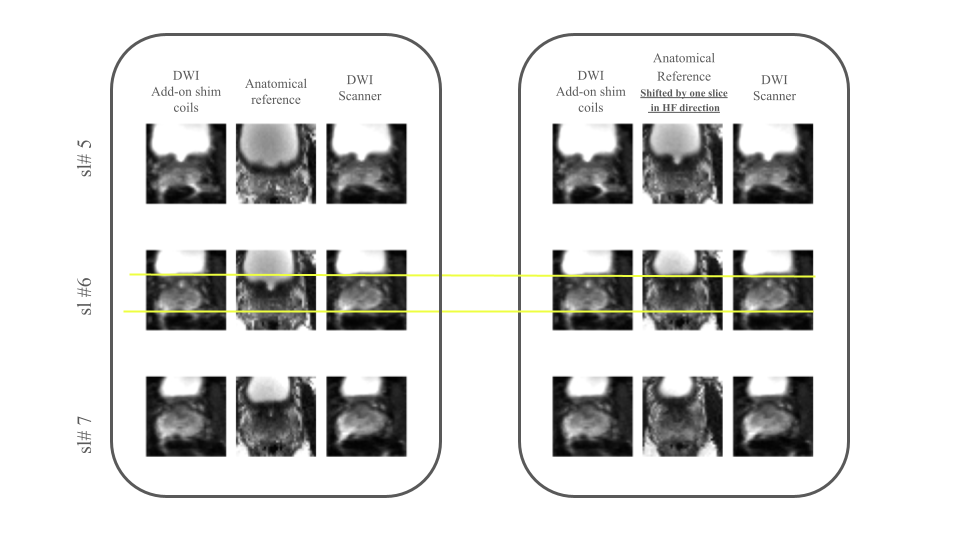


**Supp. Figure 7. Evidence of motion in head-foot direction during the DWI measurement of volunteer 7.** Three consecutive slices of the DWI and anatomical reference images are shown on the left. The same slices of the DWI image are shown on the right, but this time the anatomical reference is shifted by 1 slice in the HF direction. In the image set on the right much more accordance between anatomical features (bladder and prostate) are observed (bladder shape on slice 5, and bladder and prostate shapes on sl#6 as guided by the yellow mark lines). Note that even despite motion shimming using the add-on shims have resulted in less distortion and signal pileup in the prostate as evident in sl#7 and sl#6
